# Deletion of SH2D5 alleviates epileptic seizures and NMDAR expression via autophagic degradation of STAT1

**DOI:** 10.1172/jci.insight.191347

**Published:** 2025-08-22

**Authors:** Haokun Guo, Hui Zhang, Chenlu Zhang, Yuanyuan Shen, Liumi Jiang, Min Yang, Yuansong Zhang, Ningning Zhang, Ruirui Zhang, Ran Yu, Yong Yang, Xin Tian

**Affiliations:** 1 Department of Geriatrics,; 2 Department of Neurology, and; 3 Department of Epilepsy Center, The First Affiliated Hospital of Chongqing Medical University, Chongqing, China.; 4 Key Laboratory of Major Brain Disease and Aging Research, Ministry of Education, Chongqing Medical University, Chongqing, China.; 5 The First Clinical Medical College of Shanxi Medical University, First Hospital of Shanxi Medical University, Taiyuan, Shanxi, China.; 6 Institute for Brain Science and Disease, Chongqing Medical University, Chongqing, China.; 7 The Second Hospital & Clinical Medical School, Lanzhou University, Lanzhou, China.; 8 Department of Neurology, The Affiliated Hospital of Qingdao University, Qingdao, China.

**Keywords:** Cell biology, Neuroscience, Neurological disorders, Synapses

## Abstract

Epilepsy is a common neurological disorder resulting from an imbalance between neuronal excitation and inhibition. Synapses play a pivotal role in the pathogenesis of epilepsy. Src-homology 2 (SH2) domain–containing protein 5 (SH2D5) is highly expressed in the brain and is implicated in the regulation of synaptic function. However, its role and mechanism in epilepsy remain unclear. In this study, we found that SH2D5 was predominantly localized to pyramidal neurons in the mouse hippocampus and was upregulated in the hippocampus of epileptic brains. KO of *Sh2d5* in the hippocampus alleviated both the susceptibility to and severity of epileptic activity. Mechanistically, SH2D5 regulated N-methyl-D-aspartate receptor–mediated (NMDAR–mediated) excitatory synaptic transmission by altering the protein expression levels of NMDAR subunits. We further demonstrated that SH2D5 modulated the transcription of NMDARs by promoting the autophagic degradation of STAT1. These findings suggest that targeting the SH2D5/STAT1/NMDAR pathway may offer a potential therapeutic strategy for epilepsy.

## Abstract 2

SH2D5 affects the epileptic phenotype by regulating the autophagy of NMDARs transcription factor STAT1.

## Graphical abstract

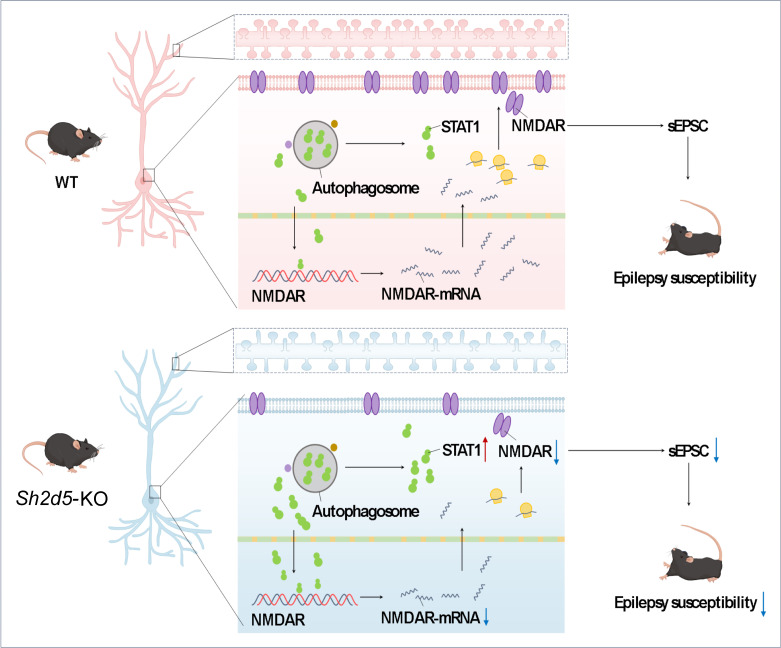

## Introduction

Epilepsy is a prevalent and debilitating neurological condition characterized by spontaneous recurrent seizures (SRSs) (1). Although more than 20 antiseizure medications (ASMs) have been developed in the last 30 years, approximately 30% of patients with epilepsy still suffer from poor seizure control. Moreover, there are no ASMs capable of preventing epileptogenesis, which is a continuous process that begins before the onset of SRSs (2). Epilepsy is thought to involve an imbalance of neuronal excitation and inhibition that originates from a local or general epileptic zone. This hypothesis is supported by the fact that many ASMs are membrane stabilizers that inhibit neural conduction, such as carbamazepine, topiramate, and lacosamide (sodium ion channel blockers), as well as synaptic transmission modulators, such as levetiracetam (binds synaptic vesicle protein 2A), valproate (elevates GABA), and perampanel (an α-amino-3-hydroxy-5-methyl-4-isoxazolepropionic acid receptor [AMPAR] antagonist). Therefore, elucidating the molecular mechanism of synaptic transmission underlying epilepsy can facilitate the exploration of potential antiepileptic targets.

SH2D5 is an adaptor-like protein with a phosphotyrosine-binding (PTB) domain at its N-terminus and an SH2-like domain at its C-terminus (3). Intracellular signal transduction relies on protein-protein interactions facilitated by specific domains, such as the SH2 and PTB domains, which recognize altered residues, such as phosphorylated tyrosine (P-Tyr) residues (4, 5). Proteins containing these domains often have additional interaction modules, enabling them to function as adapters and recruit signaling molecules to affect intracellular signaling cascades (6).

The Allen Brain Atlas revealed that SH2D5 is highly enriched within brain tissue at the transcriptional level (7). The Human Protein Atlas revealed that SH2D5 is expressed mainly in brain and testis tissues. It is expressed mainly in neurons in regions of the cerebral cortex, hippocampal formation, amygdala, and basal ganglia (8). Moreover, IHC assays revealed that, in the mouse brain, SH2D5 is enriched in Purkinje cells in the cerebellum and in pyramidal cells in the cortex and hippocampus (9). The distribution of SH2D5 underlies its presumed function in the brain. For example, a case-control study revealed that headache chronification is associated primarily with the methylation of SH2D5 at the CpG site (10). In a genome-wide association study (GWAS) on Alzheimer’s disease (AD), SH2D5 was found to be 1 of 9 genes that were differentially expressed in the brain between patients with AD and controls (11).

SH2D5 is enriched in the postsynaptic density (PSD) (9). This indicates that SH2D5 may play an important role in synaptic function. However, no studies have investigated the roles of SH2D5 in regulating synaptic transmission and in related diseases. Epilepsy is a disorder typically attributed to synaptic dysfunction. In our study, we found that SH2D5 was localized to neurons. SH2D5 was upregulated in the hippocampus in the epileptic brain, and *Sh2d5* KO alleviated epileptic activity. Analysis of synaptic function revealed that SH2D5 regulated N-methyl-D-aspartate receptor–mediated (NMDAR-mediated) excitatory synaptic transmission and the functional synapse number. Further mechanistic research revealed that SH2D5 modulated the autophagic degradation of STAT1 to affect the transcription of NMDARs. These results elucidate the role of SH2D5 in regulating epilepsy and the mechanism underlying this role.

## Results

### SH2D5 localization and expression in the epileptic brain.

**Figure 1:**
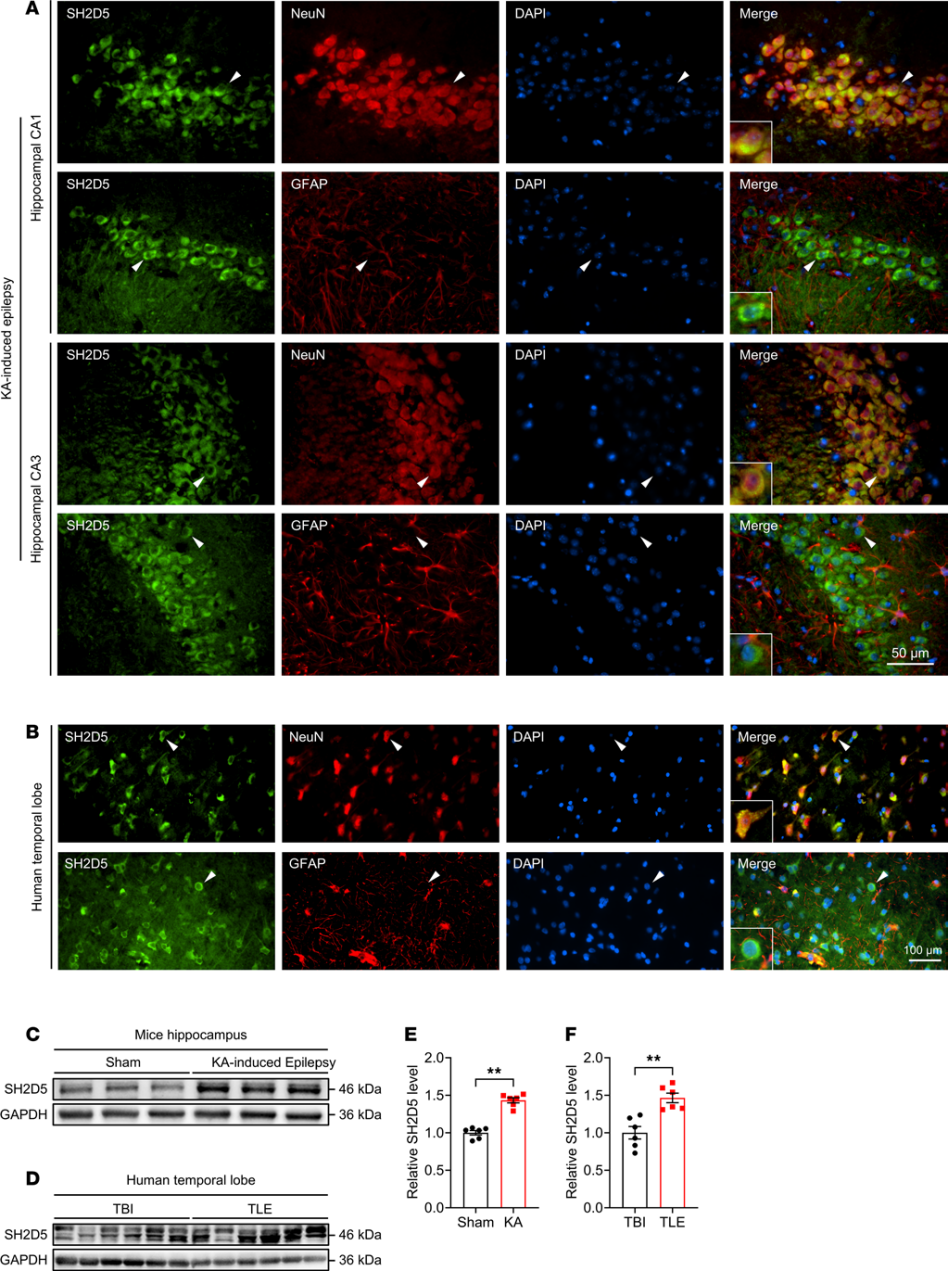
Distribution and expression of SH2D5 in epileptic brain tissues. (**A**) Representative immunofluorescence images of SH2D5 expression in mice with epilepsy. Scale bar: 50 μm. (**B**) Representative immunofluorescence images of SH2D5 expression in patients with TLE. Scale bar: 100 μm. Arrowheads: the cell that expresses SH2D5. (**C** and **E**) Representative images (**C**) and quantification (**E**) of SH2D5 expression in the hippocampi of mice with epilepsy. (**D** and **F**) Representative images (**D**) and quantification (**F**) of SH2D5 expression in patients with TLE. The data are presented as mean ± SEM. Unpaired *t* test in **E** and **F**. **P < 0.01.

To investigate the potential involvement of SH2D5 in epilepsy, we first assessed the localization of SH2D5 in epileptic brain tissues via immunofluorescence. To establish a typical epilepsy mouse model, kainic acid (KA) was used to induce status epilepticus (SE), followed by a latent period. In the hippocampi and cortex of mice with KA-induced epilepsy, SH2D5 colocalized primarily with the neuronal marker protein NeuN but not with glial fibrillary acidic protein (GFAP) (Figure 1A and Supplemental Figure 1A; supplemental material available online with this article; https://doi.org/10.1172/jci.insight.191347DS1). In temporal lobe samples obtained from patients with temporal lobe epilepsy (TLE) undergoing lesion resection, SH2D5 was also predominantly expressed in neurons (Figure 1B).

We also assessed the protein expression level of SH2D5 in epileptic brain tissues. SH2D5 protein expression in the hippocampus and cortex was elevated in mice with KA-induced epilepsy compared with control mice (Figure 1, C and E, and Supplemental Figure 1, B and C). We also detected increased expression of SH2D5 in the temporal lobe tissues of patients with TLE compared with those of control patients who experienced traumatic brain injury (TBI) but did not exhibit epilepsy symptoms (Figure 1, D and F). Detailed information on the 2 groups of patients is included in Supplemental Table 1. The change in SH2D5 expression in the epileptic brain indicates the potential involvement of SH2D5 in epilepsy.

### Sh2d5 KO alleviates epileptic activity.

**Figure 2:**
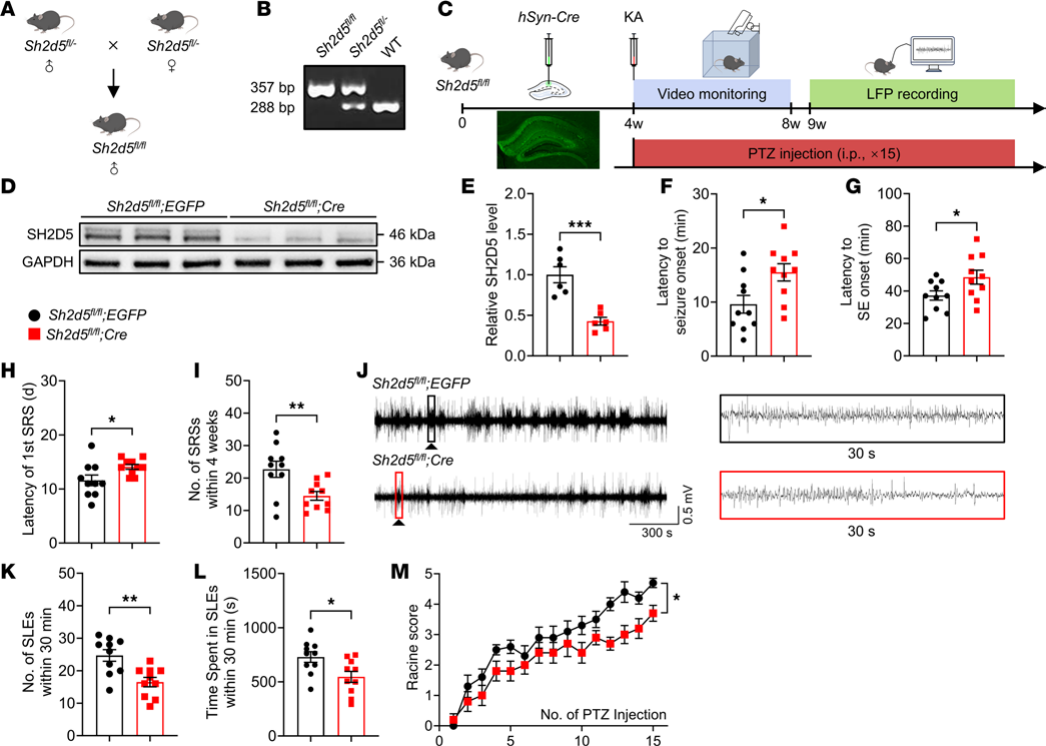
*Sh2d5* KO affects seizure susceptibility and severity. (**A**) Schematic representation of transgenic *Sh2d5* mouse breeding. (**B**) Representative image showing the genotyping of transgenic *Sh2d5* mice. (**C**) Schematic representation of the epilepsy experimental protocol. (**D** and **E**) Representative Western blot image (**D**) and quantification (**E**) of the SH2D5 protein level after the transfection of *hSyn-Cre* AAV. (**F**–**I**) Quantification of the latency to seizure onset (**F**), the latency to SE onset (**G**), the latency to the first SRS (**H**), and the total number of SRSs within 4 weeks (**I**) after KA treatment. (**J**) Representative images of LFP recordings from mice 5 weeks after KA treatment. (**K** and **L**) Quantitative analysis of LFPs. (**M**) Quantitative analysis of PTZ-induced epileptic seizures. The data are presented as mean ± SEM. Unpaired *t* test in **E**–**I**, **K**, and **L**; 2-way ANOVA with Tukey’s multiple-comparison test in **M**. *P < 0.05, **P < 0.01, and ***P < 0.001.

To investigate whether the upregulation of the SH2D5 protein contributes to the development of epilepsy, we examined its effect on epileptic activity in *Sh2d5*-KO mice. Homozygous *Sh2d5^fl/fl^* mice were obtained through the breeding of heterozygous *Sh2d5^fl/–^* mice (Figure 2A). The genotype was confirmed via PCR (Figure 2B). To specifically knock out *Sh2d5* in the neurons of the hippocampus, AAV-*hSyn-Cre* was injected into the hippocampi of *Sh2d5^fl/fl^* mice. Three weeks later, Western blotting revealed a greater-than 50% reduction in SH2D5 expression in the *Sh2d5*-KO mice (Figure 2, D and E).

A behavioral study was conducted on KA-induced epilepsy model and pentylenetetrazol (PTZ) kindling model to investigate the effect of SH2D5 on epilepsy development (Figure 2C). In the KA-induced epilepsy model, compared with control mice, *Sh2d5*-KO mice exhibited a prolonged latency to seizure onset and SE onset in the acute phase after KA administration (Figure 2, F and G). After SE onset, the brain is gradually functionally altered, leading to the generation of SRSs (12). Epileptic seizures were monitored via video recording during the first 4 weeks after SE induction, and local field potentials (LFPs) were recorded for 2 hours during the fifth week after SE induction (Figure 2C). Compared with the control group, the *Sh2d5*-KO group presented a prolonged latency to the first SRS and a reduced number of SRSs within 4 weeks (Figure 2, H and I). Moreover, LFP recording within 30 minutes revealed that *Sh2d5*-KO mice presented a significant reduction in both the duration and frequency of seizure-like events (SLEs) (Figure 2, J–L). In addition, we confirmed the effect of *Sh2d5* KO on epilepsy development in PTZ kindling model. The results revealed that *Sh2d5* KO delayed seizure kindling (Figure 2M). Taken together, these findings suggest that *Sh2d5* KO can reduce seizure susceptibility and alleviate epilepsy development.

### SH2D5 regulates excitatory synaptic transmission and the number of functional synapses.

**Figure 3:**
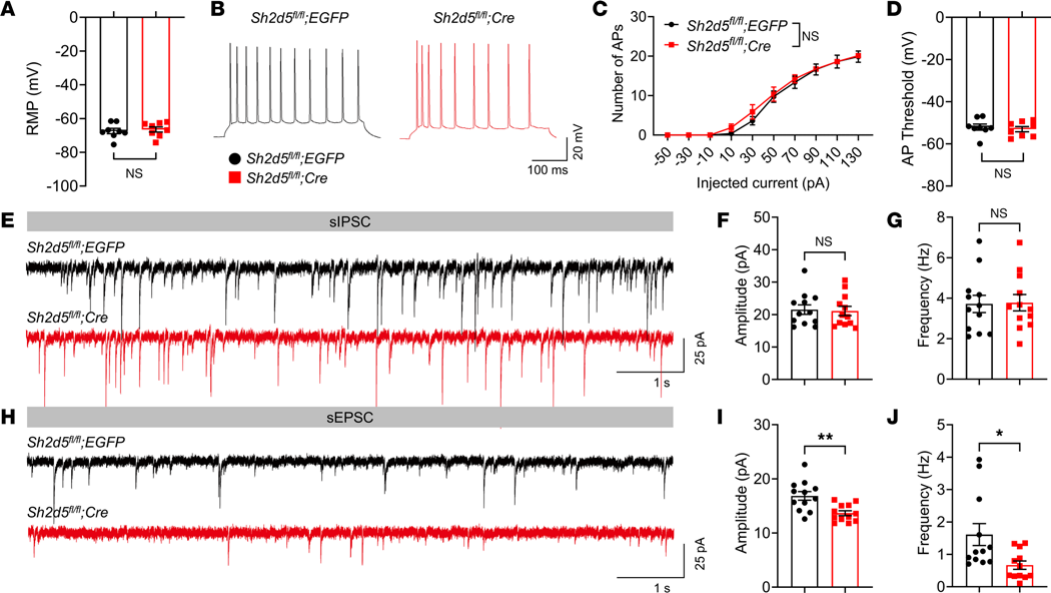
SH2D5 regulates glutamatergic synaptic transmission. (**A**) Resting membrane potential of hippocampal CA1 neurons. (**B** and **C**) Representative traces of AP spikes following current injection (90 pA) (**B**) and quantification of AP spikes in hippocampal CA1 neurons following current injection (–50 pA to 130 pA) (**C**). (**D**) Threshold for single APs evoked by a rheobase current. (**E**–**G**) Representative traces of sIPSCs of CA1 neurons (**E**) and quantification of the amplitude and frequency of sIPSCs (**F** and **G**). (**H**–**J**) Representative traces of sEPSCs of CA1 neurons (**H**) and quantification of the amplitude and frequency of sEPSCs (**I** and **J**). The data are presented as mean ± SEM. Unpaired *t* test in **A**, **D**, **F**, **G**, **I**, and **J**; 2-way ANOVA with Tukey’s multiple-comparison test in **C**. *P < 0.05, **P < 0.01.

Epilepsy is thought to result from an imbalance between excitatory and inhibitory neural activity, as well as abnormalities in intrinsic neuronal excitability (13). We utilized whole-cell patch-clamp recording to evaluate the activity of pyramidal neurons in the CA1 region. Mg^2+^ was removed from the extracellular solution of the hippocampal slices to induce seizure-like activity. We found that the resting membrane potential (RMP) (Figure 3A), action potential (AP) firing rate, and AP threshold (Figure 3, B–D) were unaltered in *Sh2d5*-KO mice, and this suggests that SH2D5 does not modulate intrinsic neuronal excitability. To elucidate the role of SH2D5 in synaptic transmission, we measured spontaneous postsynaptic currents. Our investigation revealed that the frequency and amplitude of spontaneous inhibitory postsynaptic currents (sIPSCs) were unaltered in *Sh2d5*-KO mice (Figure 3, E–G). However, *Sh2d5* KO led to a reduction in both the frequency and amplitude of spontaneous excitatory postsynaptic currents (sEPSCs) (Figure 3, H–J). Our findings suggest that SH2D5 can regulate excitatory postsynaptic transmission.

**Figure 4:**
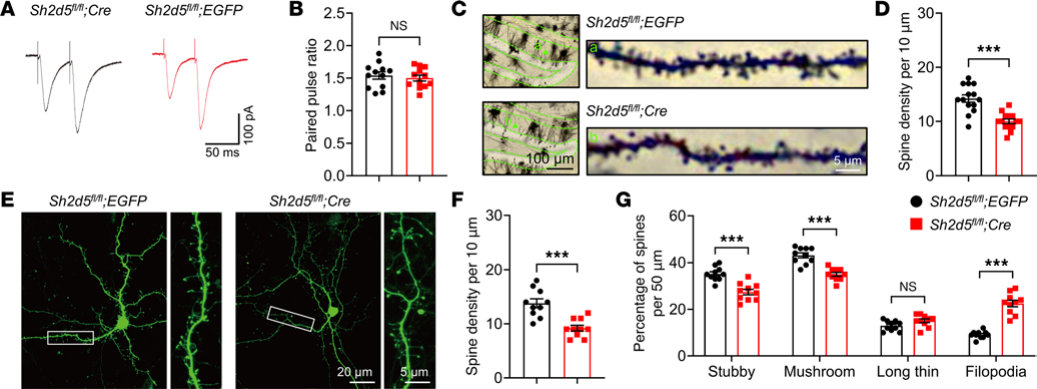
SH2D5 regulates the number of functional synapses. (**A** and **B**) Representative traces of PPR (**A**) and quantification (**B**) of the PPR. (**C** and **D**) Representative images and quantification of Golgi staining of CA1 neurons and the spine density in *Sh2d5*-KO mice. Scale bars: 100 μm (left) and 5 μm (right). (**E**) Representative immunofluorescence images of mature neurons. The images on the right are magnified at 4×. Scale bars: 20 μm (left) and 5 μm (right). (**F**) Determination of the dendritic spine density. (**G**) Quantitative analysis of dendritic spine maturity. Spines were categorized as stubby, mushroom, long thin (length > 1 μm, head diameter > 1 μm), and filopodia (length > 2 μm). Stubby and mushroom spines are mature, while long thin and filopodia spines are immature. The data are presented as mean ± SEM. Unpaired *t* tests are shown in **B**, **D**, **F**, and **G**. ***P < 0.001.

Changes in sEPSC frequency are generally attributed to changes in neurotransmitter release or the number of functional synapses (14). The paired-pulse ratio (PPR) is an indirect indicator of presynaptic release probability. We found that there was no difference in the PPR between the *Sh2d5*-KO and control groups (Figure 4, A and B). Moreover, we examined the number of functional excitatory synapses, which is correlated with parameters reflecting the morphology of dendritic spines, including the proportion of mature dendritic spines, dendritic complexity, and spine density. Golgi staining revealed that the density of dendritic spines was lower on hippocampal neurons in *Sh2d5*-KO mice than on those in control mice (Figure 4, C and D). We furtherly examined dendritic spine morphology on cultured primary neurons. Compared with those of control mice, the neurons of *Sh2d5*-KO mice exhibited not only a reduced density of dendritic spines but also significantly fewer stubby and mushroom-shaped spines, along with an increase in the number of filopodia-like protrusions (Figure 4, E–G). These findings suggest that SH2D5 can modulate the number of functional synapses.

### SH2D5 regulates NMDAR-mediated synaptic transmission.

**Figure 5:**
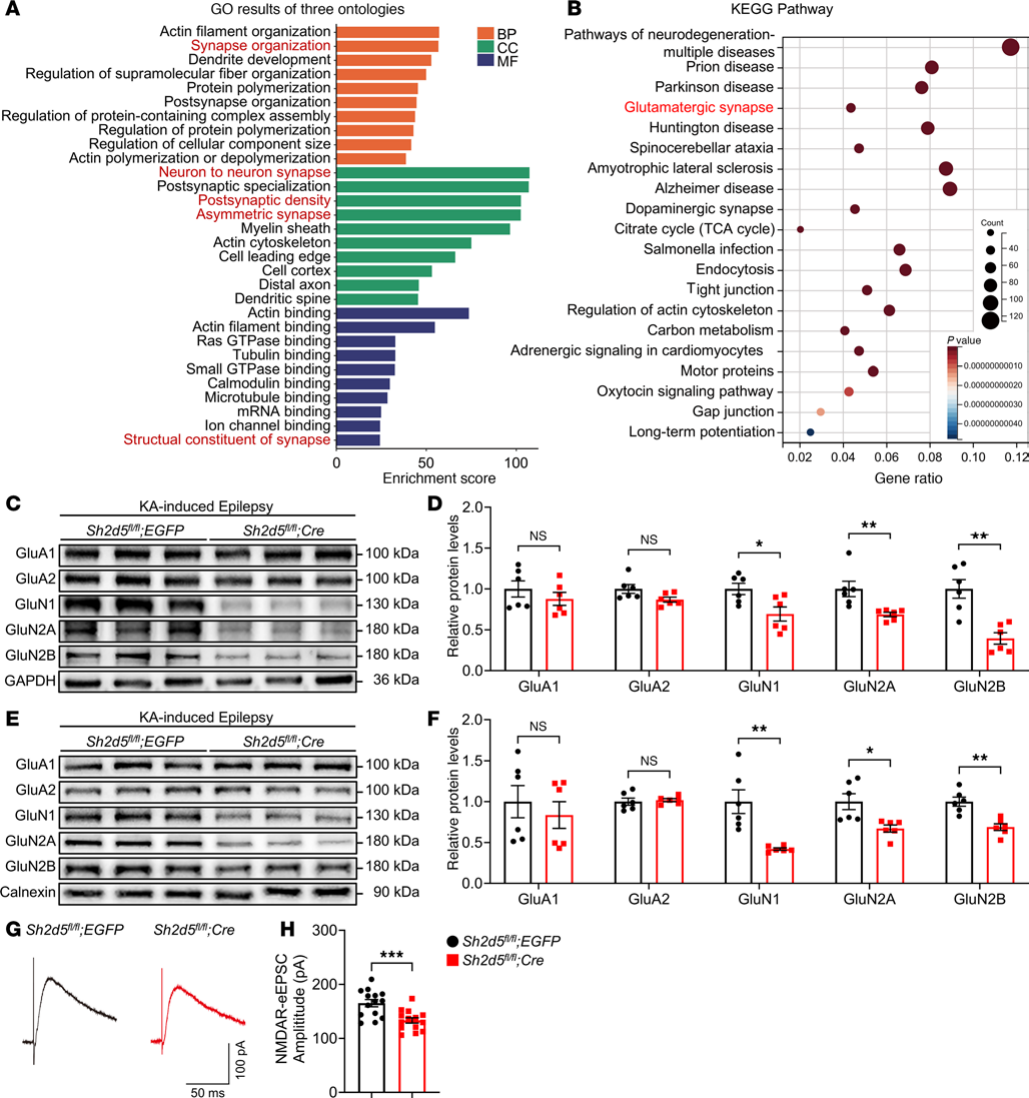
SH2D5 primarily affects the function and expression of NMDARs. (**A**) GO enrichment analysis of the IP-MS data. The proteins outlined in red are related to synapses. (**B**) KEGG pathway analysis of the IP-MS data. The proteins outlined in red are related to glutamatergic synapses. (**C** and **D**) Representative Western blot images (**C**) and quantification (**D**) of total glutamatergic receptor protein expression in the hippocampal tissue of *Sh2d5*-KO mice with epilepsy. (**E** and **F**) Representative Western blot images (**E**) and quantification (**F**) of glutamatergic receptor protein expression in the membrane fraction of hippocampal tissue from *Sh2d5*-KO mice with epilepsy. (**G** and **H**) Representative traces of NMDAR–evoked EPSCs (**G**) and quantification (**H**) of the amplitude of the NMDAR-eEPSCs. The data are presented as mean ± SEM. Unpaired *t* test in **D**, **F**, and **H**. *P < 0.05, **P < 0.01 and ***P < 0.001.

Hippocampal lysates from WT mice with KA-induced epilepsy were subjected to immunoprecipitation (IP) using an anti-SH2D5 antibody, and interacting proteins were subsequently identified by IP–mass spectrometry (IP-MS) to explore the molecular mechanisms underlying SH2D5-mediated regulation of epileptic activity and synaptic transmission. Gene Ontology (GO) enrichment analysis revealed that the potential interacting proteins were enriched primarily in functions related to synaptic activity (Figure 5A). Kyoto Encyclopedia of Genes and Genomes (KEGG) pathway analysis revealed that the potential interacting proteins were associated with glutamatergic synapses (Figure 5B). These findings support the electrophysiological findings that SH2D5 regulates excitatory synaptic transmission. Furthermore, to determine the type of excitatory synaptic receptor modulated by SH2D5, the protein levels of AMPAR and NMDAR subunits in epileptic hippocampal tissue were assessed. Western blotting demonstrated that *Sh2d5* KO resulted in a significant decrease in the total protein levels of the GluN1, GluN2A, and GluN2B subunits in epileptic hippocampal tissue. However, *Sh2d5* KO had no discernible effect on the total protein levels of AMPAR subunits (Figure 5, C and D). Furthermore, given that membrane proteins play roles in synaptic transmission, the membrane fraction of hippocampal samples was extracted, and the levels of synaptic receptor subunits were measured. NMDAR subunit expression in the membrane fraction of hippocampal tissues from *Sh2d5*-KO mice was reduced, whereas no significant change in AMPAR subunit expression was detected (Figure 5, E and F). Functionally, we observed a reduction in the amplitude of NMDAR–evoked EPSCs (NMDAR-eEPSCs) in hippocampal slices from *Sh2d5*-KO mice (Figure 5, G and H). Taken together, these findings suggest that SH2D5 regulates NMDAR-mediated synaptic transmission in epilepsy.

### STAT1 mediates the SH2D5-induced regulation of NMDAR expression and epileptic activity.

**Figure 6:**
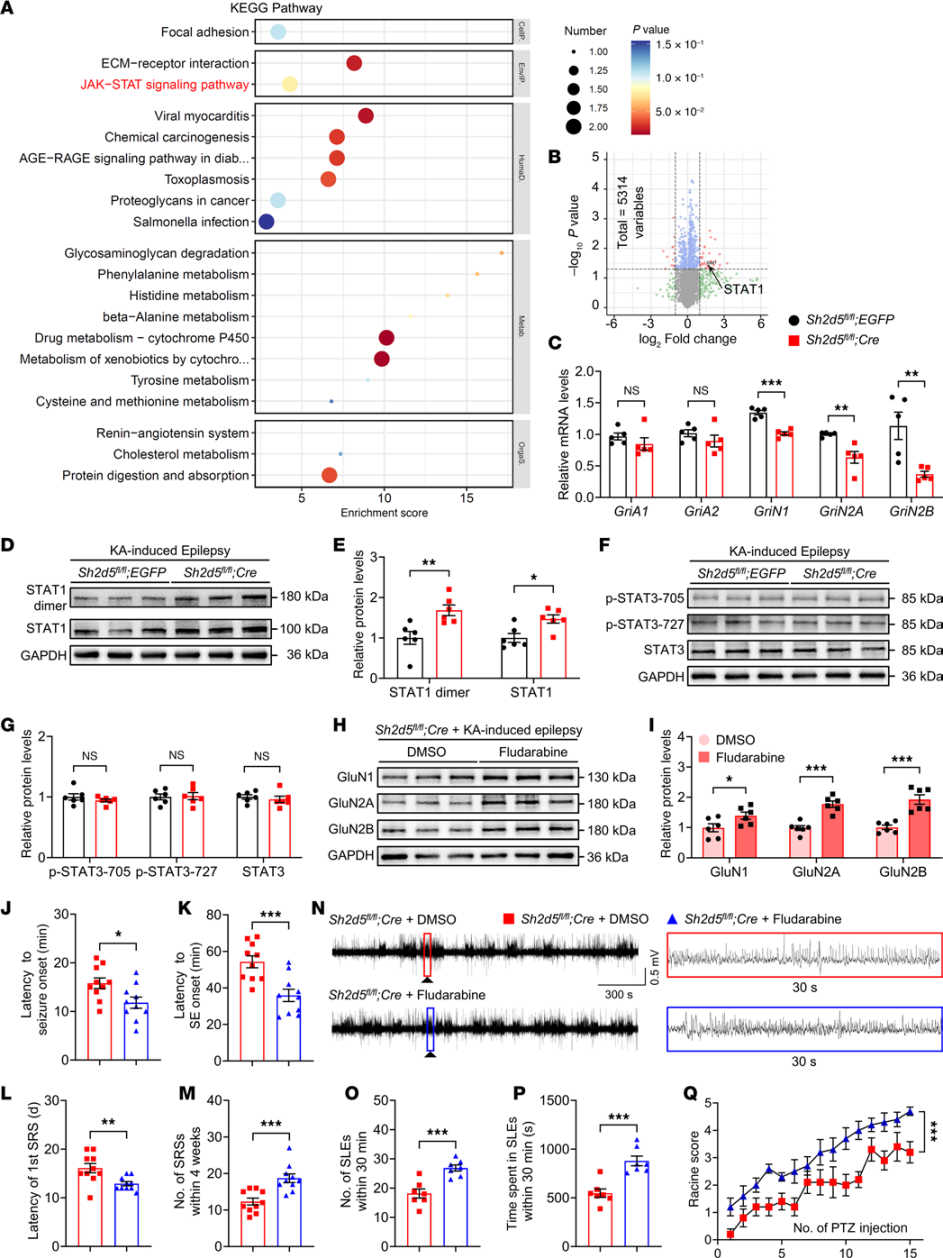
SH2D5 modulates NMDAR transcript levels via STAT1. (**A**) KEGG pathway analysis of the LC–MS/MS quantitative proteomics data from *Sh2d5*-KO and control mice with epilepsy. The proteins outlined in red are related to the JAK/STAT signaling pathway. (**B**) Volcanic map of differentially expressed proteins identified by LC-MS/MS quantitative proteomics. (**C**) Verification of changes in glutamatergic receptor levels via qPCR. (**D** and **E**) Representative Western blot images (**D**) and quantification (**E**) of STAT1 and STAT1 dimer levels. (**F** and **G**) Representative Western blot images (**F**) and quantification (**G**) of p-STAT3-705, p-STAT3-727, and STAT3 levels. (**H** and **I**) Representative Western blot images (**H**) and quantification (**I**) of the protein levels of NMDARs after inhibition of STAT1 by fludarabine. (**J**–**M**) Quantification of the latency to seizure onset (**J**), latency to SE onset (**K**), latency to the first SRS (**L**), and total number of SRSs within 4 weeks (**M**) after KA treatment. (**N**) Representative images of LFP recordings taken 5 weeks after KA treatment. (**O** and **P**) Quantitative analysis of LFP recordings. (**Q**) Quantification of PTZ kindling–induced epileptic seizures. The data are presented as mean ± SEM. Unpaired *t* test in **C**, **E**, **G**, **I**–**M**, **O**, and **P**; 2-way ANOVA with Tukey’s multiple-comparison test in **Q**. *P < 0.05, **P < 0.01, and ***P < 0.001.

To explore how SH2D5 regulates NMDAR expression, hippocampal proteins were extracted from *Sh2d5* KO and control mice, both subjected to KA-induced epilepsy, and subjected to liquid chromatography–tandem MS (LC–MS/MS) analysis. In total, 5,314 distinct proteins were identified. KEGG pathway analysis revealed that the differentially expressed proteins were related to various pathways (Figure 6A). We specifically focused on the JAK/STAT pathway because numerous reports have highlighted its potential role in the transcriptional regulation of NMDARs (15). According to a volcano plot, the protein STAT1 of the JAK/STAT pathway was specifically differentially expressed between *Sh2d5*-KO mice and control mice (Figure 6B). To confirm the interaction between SH2D5 and STAT1, we assessed their colocalization via fluorescence staining and their interactions via co-IP analysis (Supplemental Figure 2, A–C). Furthermore, as STAT1 has been reported to regulate the transcription of NMDARs (16), we examined the transcriptional levels of NMDARs. Similar to the Western blotting results shown in Figure 5, C and D, quantitative PCR (qPCR) analysis revealed a significant decrease in the mRNA level of NMDARs in *Sh2d5*-KO mice with epilepsy (Figure 6C), suggesting that SH2D5 may modulate the transcription of NMDARs. Functionally, the protein levels of STAT1 and dimerized STAT1 were increased in the hippocampi of *Sh2d5*-KO mice with epilepsy (Figure 6, D and E). STAT3, a member of the STAT protein family, has been reported to regulate NMDAR transcription (17) and is regulated by SH2D5 in hepatocellular carcinoma (18). We also evaluated the expression of both total and phosphorylated STAT3 in *Sh2d5*-KO and control mice. No significant differences in total STAT3 protein, p-705-STAT3, or p-727-STAT3 levels were observed (Figure 6, F and G). These findings indicate that SH2D5 may be involved in NMDAR transcription through STAT1.

To further confirm that STAT1 functionally participates in the regulation of epilepsy by SH2D5, we performed a rescue experiment in which STAT1 was inhibited with fludarabine. We found that i.p. injection of fludarabine resulted in a significant increase in the protein expression of NMDARs in the hippocampi of *Sh2d5*-KO mice with KA-induced epilepsy (Figure 6, H and I). A behavioral study revealed that *Sh2d5*-KO mice treated with fludarabine presented a shorter latency to seizure onset, SE onset, and the first SRS (Figure 6, J–L) and an increased number of SRSs (Figure 6M) than did those treated with dimethyl sulfoxide (DMSO). LFP analysis of mice with KA-induced epilepsy revealed that *Sh2d5*-KO mice treated with fludarabine presented significant increases in the frequency of SLEs and the duration of SLEs (Figure 6, N–P). In the PTZ kindling epilepsy model, the epileptic activity of the mice in the fludarabine group was greater than that of the mice in the control group (Figure 6Q). Collectively, these findings suggest that STAT1 plays an important role in the regulation of epileptic activity by SH2D5.

### SH2D5-mediated autophagy modulates the expression of STAT1.

**Figure 7:**
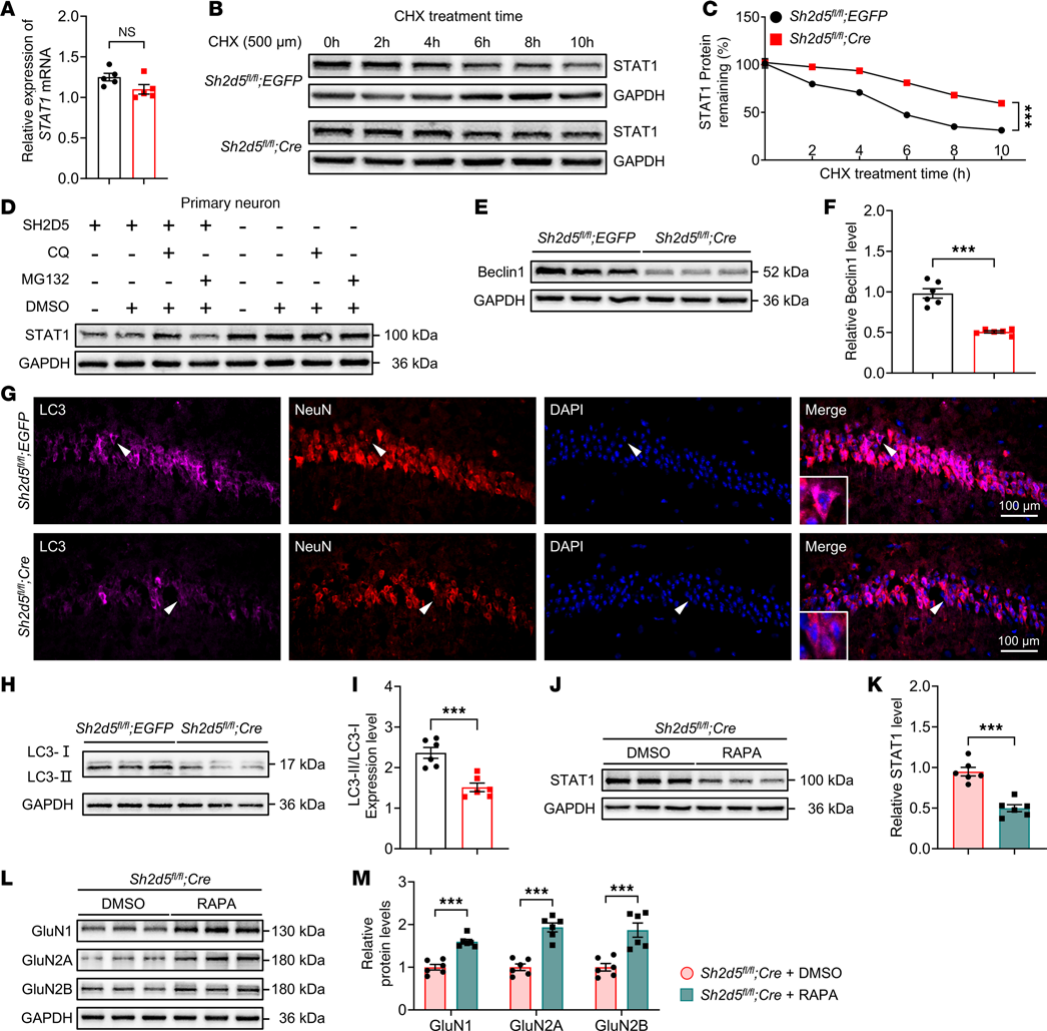
SH2D5-mediated autophagy modulates the expression of STAT1. (**A**) Quantification of *STAT1* mRNA levels via qPCR. (**B** and **C**) Representative Western blot images (**B**) and quantification (**C**) of the protein level of STAT1 at different timepoints after CHX treatment. (**D**) Western blot images showing the STAT1 protein levels in the CQ, MG132, and DMSO treatment groups. (**E** and **F**) Representative Western blot images (**E**) and quantification (**F**) of the protein level of Beclin1. (**G**) Representative immunofluorescence images showing SH2D5 and LC3 colocalization. Scale bar: 100 μm. Arrowheads: the cell that expresses LC3. (**H** and **I**) Representative Western blot images (**H**) and quantification (**I**) of LC3-II/LC3-I levels. (**J** and **K**) Representative Western blot images (**J**) and quantification (**K**) of STAT1 levels after RAPA administration. (**L** and **M**) Representative Western blot images (**L**) and quantification (**M**) of GluN1, GluN2A, and GluN2B levels after RAPA administration. The data are presented as mean ± SEM. Unpaired *t* test in **A**, **F**, **I**, **K**, and **M**; 2-way ANOVA with Tukey’s multiple-comparison test in **C**. ***P < 0.001.

As shown above, *Sh2d5* KO upregulated STAT1 protein expression levels; therefore, we next explored the mechanism by which SH2D5 regulates STAT1 protein levels. We first investigated whether SH2D5 regulates the mRNA expression of STAT1 in brain tissue from epileptic mice via qPCR. The absence of SH2D5 did not significantly affect the mRNA level of STAT1 (Figure 7A). Next, we investigated the effect of SH2D5 on the posttranscriptional stability of STAT1. Primary hippocampal neurons from *Sh2d5^fl/fl^* mice were transfected with *AAV-hSyn-Cre*. A cycloheximide (CHX) chase assay was used to assess the stability of STAT1. Our results revealed that *Sh2d5* KO significantly prolonged the half-life of STAT1 (Figure 7, B and C), indicating that SH2D5 may regulate STAT1 protein degradation. To confirm these results, we investigated the degradation of STAT1 by the 2 most common degradation pathways — the ubiquitination and lysosomal pathways. We observed that chloroquine (CQ; a lysosomal inhibitor) significantly increased STAT1 protein levels, whereas the administration of MG132 (a ubiquitination inhibitor) did not (Figure 7D). In addition, *Sh2d5* KO decreased the levels of the autophagy-related protein Beclin1 and the LC3-II/LC3-I ratio (Figure 7, E, F, H, and I). Immunofluorescence staining also revealed a significant reduction in the mean fluorescence intensity of LC3 relative to that of NeuN in the hippocampal CA1 region in *Sh2d5*-KO mice (Figure 7G). A rescue experiment in which *Sh2d5*-KO mice were treated with the autophagy agonist RAPA revealed a notable reduction in the protein level of STAT1 (Figure 7, J and K) and a significant increase in the protein expression of NMDARs (Figure 7, L and M), which further confirmed the role of SH2D5 in regulating autophagy. To date, the mechanism of SH2D5 regulating autophagy has not been reported. Previous studies have shown that knockdown of SH2D5 reduced the level of RAC1-GTPase (9), and others have suggested that autophagy can be increased through the RAC1 (19, 20). Therefore, we checked the role of RAC pathway agonist SEW2871 in *Sh2d5*-KO mice. We found that SEW2871 significantly increased the LC3II/LC3I ratio and the expression level of Beclin1 in the hippocampal tissue, whereas it reduced the level of STAT1 protein (Supplemental Figure 2, D–G). Taken together, these findings suggest that SH2D5 regulates STAT1 expression via the modulation of autophagy.

## Discussion

This study investigated the role of SH2D5 in epilepsy and the mechanisms underlying this role. Our findings show that SH2D5 is mainly localized in hippocampal pyramidal neurons. Using *Sh2d5^fl/fl^* mice and *hSyn-Cre* AAV, we selectively knocked out *Sh2d5* in adult neurons. Neuronal *Sh2d5* KO reduced the severity of SRSs and seizure susceptibility. IP-MS and LC–MS/MS analyses identified STAT1 as a key downstream target of SH2D5. SH2D5 was found to regulate the transcriptional repression of Grin1, Grin2a, and Grin2b by controlling STAT1 degradation via selective autophagy. KO of *Sh2d5* increased STAT1 levels, inhibited NMDAR transcription, and alleviated epilepsy symptoms, while inhibiting STAT1 worsened seizures. These results provide additional insights into the role of SH2D5 in epilepsy.

We verified via whole-cell patch-clamp recording that the deletion of SH2D5 had an effect on sEPSCs. We used IP-MS to identify proteins whose expression is modulated by SH2D5 in the context of epilepsy and revealed that SH2D5 interacts with a multitude of synapse-associated proteins, particularly those in the glutamatergic synaptic pathway. AMPARs and NMDARs are the predominant types of glutamate receptors responsible for transmitting signals from presynaptic neurons to postsynaptic neurons, and their localization, function, and abundance are tightly regulated (21, 22). Changes in their expression and function are closely linked to epilepsy (23, 24). In our study, we observed a significant decrease in the levels of NMDAR proteins (GluN1, GluN2A, and GluN2B) in both the total fraction and the membrane fraction of hippocampal tissue following *Sh2d5* KO. These findings suggest that the modulation of NMDAR protein expression by SH2D5 could have an effect on synaptic activity in epilepsy.

NMDARs are crucial for mediating synaptic transmission and plasticity (25). Deletion of SH2D5 did not result in significant changes in the PPR but did alter the number and complexity of dendritic spines, suggesting that the probability of presynaptic glutamate release was unchanged but that synaptic remodeling was altered in epilepsy. As functional excitatory synapses are predominantly located at dendritic spines (24), the number of excitatory synapses is associated with the morphology of dendritic spines, including the proportion of mature dendritic spines, dendritic spine complexity, and spine density. Therefore, SH2D5 modulates the function of excitatory synapses by altering the morphology of dendritic spines, affecting behavior and epileptiform-related phenotypes in epileptic mice, which is consistent with the findings obtained from patch-clamp recordings.

Our findings suggest that STAT1 is the key gene regulated by SH2D5 in mice with epilepsy. The STAT family consists of 7 members, specifically STAT1, STAT2, STAT3, STAT4, STAT5a, STAT5b, and STAT6 (26). These proteins play pivotal roles in regulating fundamental physiological processes, such as growth, division, and apoptosis (27). Under physiological conditions, STAT1 is present in an inactive state within the cytoplasm. Upon activation by cytokines or growth factors, STAT1 undergoes dimerization and translocates to the nucleus, where it binds to DNA to modulate gene transcription (28, 29). Subsequently, activated STAT1 can directly bind to NMDAR promoters to repress NMDAR expression (16). STAT3, another member of the STAT protein family, has been demonstrated to be involved in the regulation of epilepsy (30). Moreover, STAT3 increases NMDAR transcription through direct binding to a specific Gamma-activated site (GWS) in NMDAR promoters (17). STAT3 is primarily activated via phosphorylation at the tyrosine 705 (Y705) or serine 727 (S727) residue (31). However, no significant changes in the protein levels of STAT3, p-STAT3-705, or p-STAT3-727 were detected. Finally, we confirmed a significant increase in NMDAR expression following the administration of the STAT1 inhibitor fludarabine, indicating that SH2D5 regulates NMDAR expression through STAT1.

The maintenance of protein homeostasis and the regulatory mechanisms involved in protein synthesis and degradation are essential for the normal functioning of the central nervous system (32, 33). Autophagy, a crucial process for the clearance of abnormal proteins, plays dual roles in various diseases, such as diabetic retinopathy, ischemic stroke, and epilepsy (34, 35). Our experimental results also confirmed that SH2D5-mediated autophagy is responsible for the degradation of the STAT1 protein. Owing to the limited research on the function of the SH2D5 protein, there are no reports regarding the association between SH2D5 and autophagy. However, numerous proteins containing SH2 domains are capable of modulating autophagy levels. For example, methyl vinyl ketone can modulate autophagy by binding to the SH2 domain of phosphatidylinositol-3 kinase (36), and the SH2 supercomplex has been reported to inhibit autophagy in pancreatic ductal adenocarcinoma cells (37). However, our results indicate that *Sh2d5* KO negatively regulates autophagy, which is contrary to previously reported effects. Therefore, we speculate that, in the hippocampi of epileptic mice, SH2D5 may not directly regulate autophagy through its SH2 domain. Additionally, studies have shown that knockdown of SH2D5 reduced the level of RAC1-GTPase (9) and that RAC1 affects autophagy (19, 20). The level of autophagy was significantly increased after the use of RAC pathway agonists, while the level of STAT1 protein was significantly decreased. These results further support the hypothesis that SH2D5 may regulate autophagy through the RAC1 signaling pathway, thereby regulating STAT1 protein levels.

These studies indicate a potential association between the expression of SH2D5 and the process of autophagy. Finally, we demonstrated through rescue experiments that the level of the STAT1 protein was significantly reduced and that the protein levels of NMDARs were significantly elevated following the administration of the autophagy agonist RAPA. These findings suggest that reliance on SH2D5-mediated neuronal autophagy contributes to accelerating the onset of epilepsy.

In conclusion, our findings suggest that SH2D5 regulates autophagy in hippocampal neurons in the brain, thereby modulating the expression of STAT1 and subsequently influencing the transcription of NMDARs, ultimately affecting epilepsy-related behavioral phenotypes. These observations provide additional insights into the pathogenesis of epilepsy and identify potential targets for therapeutic interventions.

## Methods

### Sex as a biological variable.

The human brain samples used in this work were collected from male and female individuals (Supplemental Table 1). Our study exclusively examined male mice to reduce female sexual cycle–related variation. It is unknown whether the findings in male mice are relevant to female mice.

### Animals.

*Sh2d5-*Flox mice (NM-CKO-226053) on a C57BL/6J background were obtained from the Shanghai Model Organisms Center (Shanghai, China). The WT male C57BL/6J mice were procured from the Experimental Animal Center of Chongqing Medical University. All animals were maintained and bred under specific pathogen–free conditions with ad libitum access to food and water on a 12-hour light/dark cycle at a controlled temperature and humidity. Male *Sh2d5^fl/fl^* mice at 7–8 weeks of age were treated with *hSyn-Cre* AAV for subsequent experiments. Primary neurons were isolated from the hippocampi of neonatal *Sh2d5^fl/fl^* mice (both males and females) and cultured. On day 3, the neurons were treated with *hSyn-Cre* AAV.

### Intrahippocampal injection of AAV vectors.

To obtain *Sh2d5*-KO mice, *Sh2d5^fl/fl^* mice were anesthetized with isoflurane (3%–5% for induction, 1%–2% for maintenance) and fixed on a stereotaxic apparatus. rAAV-*hSyn*-Cre-EGFP-WPRE-hGH pA (2 × 10^12^ TU/mL) and rAAV-*hSyn*-EGFP-WPRE-hGH pA (2 × 10^12^ TU/mL) were obtained from BrainVTA Company. The AAVs (0.2 μL) were bilaterally injected into the dorsal hippocampus via a 0.5 μL microsyringe (Hamilton Bonaduz). The syringe was maintained in situ for 10 minutes to prevent reflux. After 1 month, the mice in the rAAV-*hSyn*-EGFP (*Sh2d5^fl/fl^;EGFP*) and rAAV-*hSyn*-Cre-EGFP (*Sh2d5^fl/fl^;Cre*) groups were used for animal experiments.

### TLE mouse model.

Male C57BL/6J mice aged 8 weeks (22–24 g) were anesthetized with isoflurane (3%–5% for induction, 1%–2% for maintenance) and secured on a stereotaxic apparatus. A total of 1.0 nmol KA (50 nL) was injected into the right hippocampus over a period of 1 minute via a 0.5 μL microinjector (coordinates from bregma: anteroposterior [AP], −1.5 mm; mediolateral [ML], −1.5 mm; dorsoventral [DV], −1.5 mm). The microinjector was kept in place for an additional 2 minutes to minimize backflow. Two hours following the onset of SE, diazepam (5 mg/kg) was administered to terminate seizure activity. After they recovered from diazepam treatment, the mice were individually housed and monitored for seizure development. After termination of acute SE episodes, the mice were monitored for 4 weeks, and the number of SRSs was evaluated; SRSs greater than grade 3 according to the Racine scale were included for behavioral analysis.

The severity of seizures was classified according to the Racine scale as follows: grade 0: normal behavior and no abnormality; grade 1: immobilization, lying on the belly; grade 2: head nodding, facial, forelimb, or hindlimb myoclonus; grade 3: continuous whole-body myoclonus, myoclonic jerks, or holding the tail up stiffly; grade 4: rearing, tonic seizure, or falling down on its side; and grade 5: tonic-clonic seizure, falling down on its back, wild rushing and jumping, or death.

### LFP recordings.

For LFP recordings, a bipolar recording electrode was implanted in the dorsal aspect of the right hippocampus (coordinates from bregma: AP, −1.6 mm; ML, −1.6 mm; DV, −1.5 mm), and 2 stainless steel screws were also implanted in the frontal lobe and attached to the skull as ground screws. Five weeks after KA-induced SE, LFP data were acquired and analyzed via a 3-channel tethered EEG system (Pinnacle Technology) with Sirenia Acquisition software v2.2.1 (Pinnacle Technology). A 30-minute baseline recording was obtained prior to each session. LFP recordings were conducted over a 3-day period, during which the movement of the mice was unrestricted and the mice had ad libitum access to food and water. Seizures were identified by characteristic peak wave discharges on LFPs.

The criteria for SLEs were derived from previously published guidelines (38). In brief, a convulsion was defined as a sudden change in amplitude > 2 SDs from the baseline mean, as this threshold is sufficient to distinguish seizures from normal LFPs. The baseline mean was calculated from data recorded 30 seconds before a possible seizure, with the duration of the data being greater than 5 seconds.

### PTZ kindling model.

PTZ kindling was conducted between 9:00 a.m. and 12:00 p.m. The weights of the animals were measured, and PTZ was i.p. injected every other day at a dose of 35 mg/kg. The behavior of the animals was observed within 30 minutes after PTZ injection, and the seizure grade was evaluated according to the Racine scale. When the seizure grade of a mouse reached 4–5 after 3 consecutive injections, kindling was considered successful.

### Drug application in animal models.

To determine the effects of SH2D5-independent autophagic degradation on STAT1, after KA treatment, the mice were i.p. injected with 0.8 mg/kg fludarabine (MedChemExpress), 2.0 mg/kg RAPA (MedChemExpress), or an equal volume of corn oil every other day for 28 days. At the same time, in order to detect whether SH2D5 regulates autophagy through RAC1, we also i.p. injected 0.5 mg/kg SEW2871 or an equal volume of DMSO once a day for 2 weeks.

### Mouse brain tissue preparation.

For Western blotting, PCR, and co-IP, the mice were anesthetized by i.p. injection of 100 mg/kg pentobarbital and sacrificed. Hippocampal tissue was collected and either used immediately or frozen in liquid nitrogen for future use.

For immunofluorescence analysis, the mice were perfused with 30 mL of 1× phosphate-buffered saline (PBS), followed by 30 mL of 4% paraformaldehyde (PFA). The brains were fixed in 4°C PFA for 24 hours, treated with 20% sucrose for 18 hours and 30% sucrose for 36 hours for cryoprotection, and then sectioned. The brain tissues were embedded in optimal cutting temperature compound and frozen in isopentane on dry ice. The tissues were subsequently sectioned into 10 mm coronal sections using a Leica cryostat (Wetzlar). The brain tissue sections were stored at –80°C until they were used for immunofluorescence analysis.

### qPCR.

Total RNA was isolated from cortical and hippocampal tissues via TRIzol reagent following the protocol provided by the manufacturer (Invitrogen). The concentration and purity of the total RNA were assessed via a Nanodrop 2000 spectrophotometer. cDNA was subsequently synthesized from 600 ng of RNA via HiScript II Q Select RT SuperMix for qPCR (Vazyme Biotech) according to the manufacturer’s instructions. The total reaction volume for cDNA synthesis was 20 μL. For real-time PCR analysis, PCR was performed with 1 μL of cDNA template using the SYBR Green qPCR Master Mix Kit (Vazyme Biotech) on a StepOnePlus Real-Time PCR system (Applied Biosystems). GAPDH served as the reference gene. Each sample was analyzed in triplicate or duplicate, and gene expression levels were quantified as fold changes via the comparative Ct method (2^−ΔΔCt^). The primer sequences are listed in Supplemental Table 2.

### Human brain tissue.

Human brain tissue samples from patients with TLE or TBI who underwent temporal lobectomy were obtained from the First Affiliated Hospital of Chongqing Medical University.

### Western blotting.

Mouse hippocampal tissue and primary neurons were collected for Western blotting and immunofluorescence analysis. Plasma membrane protein extraction was performed following the manufacturer’s instructions (Solarbio). The protein concentration was quantified using the bicinchoninic acid (BCA) method (Beyotime Biotechnology). Equal amounts of protein were first separated via SDS-PAGE and then electrotransferred to a polyvinylidene difluoride membrane (MilliporeSigma). The membrane containing the protein samples was then incubated with rapid blocking solution (Beyotime Biotechnology) for another 15 minutes at room temperature (RT). The membranes were incubated overnight with primary antibodies diluted in primary antibody dilution buffer (Beyotime Biotechnology), washed 3 times with TBST, and subsequently incubated with diluted secondary antibodies for 1 hour at RT. After the secondary antibody incubation, the membranes were washed 3 times with TBST. Finally, the membranes were incubated with enhanced chemiluminescence reagent (Biosharp), and the signals were detected with a Fusion FX7 image analysis system (Vilber Lourmat). Primary antibodies against the following proteins were used for Western blotting: anti-SH2D5 rabbit antibody (1:1,000; PA5-101883, Thermo Fisher Scientific), anti-STAT1 rabbit antibody (1:2,000; 10144-2-AP, Proteintech), anti-STAT3 rabbit antibody (1:1,000; 10253-2-AP, Proteintech), anti–p-STAT3-705 rabbit antibody (1:1,000; EPR23968-52, Abcam), anti–p-STAT3-727 rabbit antibody(1:1,000; E121-31, Abcam), anti-GAPDH rabbit antibody (1:4,000; 10494-1-AP, Proteintech), anti-Calnexin rabbit antibody (1:2,000; 10427-2-AP, Proteintech), anti-GluA1 rabbit antibody(1:2,000; 25012-1-AP, Proteintech), anti-GluA2 rabbit antibody(1:2,000; 11994-1-AP, Proteintech), anti-GluN1 rabbit antibody (1:2,000; 27676-1-AP, Proteintech), anti-GluN2A rabbit antibody (1:2,000; 28571-1-AP, Proteintech), anti-GluN2B rabbit antibody (1:1,000; 21920-1-AP, Proteintech), anti-Beclin1 rabbit antibody (1:2,000; sc-48341, Santa Cruz Biotechnology), and anti-LC3 rabbit antibody (1:2,000; 18725-1-AP, Proteintech).

### Immunofluorescence analysis.

For immunofluorescence staining of cryostat sections, the frozen sections were first warmed to RT. Antigen retrieval was then performed with sodium citrate solution, and the sections were permeabilized with 0.4% Triton X-100 for 20 minutes, blocked with goat serum (Boster Biological Technology) for 60 minutes at RT, and incubated with mixed primary antibodies diluted in PBS at 4°C overnight. After being washed 3 times with PBS, the sections were incubated with fluorescence-conjugated secondary antibodies for 60 minutes at RT in the dark. The nuclei were stained with DAPI and then coverslips were applied for mounting.

For immunocytochemical staining, primary neurons were fixed in a PBS solution containing 4% PFA and 4% sucrose for 30 minutes at RT. The neurons were permeabilized with 0.1% Triton X-100 for 10 minutes and blocked with goat serum for 60 minutes at RT. The samples were then incubated with mixed primary antibodies diluted in PBS at 4°C overnight. After being washed 3 times with PBS, the neurons were incubated with fluorescent secondary antibodies for 60 minutes at RT in the dark. All images were captured using a confocal microscope (Leica). Primary antibodies against the following proteins were used for immunofluorescence analysis: anti-SH2D5 rabbit antibody (1:200; PA5-101883; Thermo Fisher Scientific), anti-NeuN mouse antibody (1:200; MAB377; MilliporeSigma), anti-GFAP mouse antibody (1:200; 60190-1-Ig; Proteintech), and anti-LC3 mouse antibody (1:100; 66139-3-Ig; Proteintech).

### IP-MS and LC-MS/MS analysis.

Total protein was extracted from hippocampal tissue samples 28 days after KA injection. Anti-SH2D5 antibody–bound beads were used to immunoprecipitate proteins that interact with SH2D5. The interacting proteins were separated by SDS-PAGE. After electrophoresis, the cells were stained with Coomassie brilliant blue for 30 minutes at RT. For MS analysis, we excised the lanes without protein bands on the gel. IP-MS was performed by Applied Protein Technology Co.

Twenty-eight days after Cre-AAV injection into the hippocampus, the mice were divided into the *Sh2d5^fl/fl^;EGFP* and *Sh2d5^fl/fl^;Cre* groups and then treated with KA for epilepsy modeling. After 28 days, hippocampal protein was extracted from the 2 groups of mice (*n* = 4). Protein extraction, digestion, SDS-PAGE, LC-MS/MS analysis, protein identification, quantification, and bioinformatics analysis were performed by Applied Protein Technology Co.

### Whole-cell patch-clamp recordings.

After the mice were anesthetized and decapitated, the brain tissue was promptly isolated in ice-cold slice solution (containing 2.5 mM KCl, 1.25 mM NaH_2_PO_4_, 6 mM MgCl_2_, 1 mM CaCl_2_, 26 mM NaHCO_3_, 220 mM sucrose, and 10 mM D-glucose; bubbled with a mixture of 95% O_2_ and 5% CO_2_) and then sectioned into 300 μm slices using a vibratome (Leica VT1200S) in the same ice-cold slice solution. The slices were subsequently equilibrated in artificial cerebrospinal fluid (ACSF; composed of 3 mM KCl, 124 mM NaCl, 2 mM CaCl_2_, 2 mM MgCl_2_, 1.23 mM NaH_2_PO_4_, 26 mM NaHCO_3_, and 10 mM D-glucose; bubbled with 95% O_2_ and 5% CO_2_) at 32°C for 1 hour. The slices were fully submerged in flowing Mg^2+^-free ACSF (3 mL/min) at RT, and recordings were obtained using a Multiclamp 700B amplifier (Axon).

For current-clamp recordings, the internal mixture consisted of 60 mM K_2_SO_4_, 60 mM NMG, 12 mM phosphocreatine, 40 mM HEPES, 4 mM MgCl_2_, 0.5 mM BAPTA, 2 mM Na_2_ATP, and 0.2 mM Na_3_GTP. A current step protocol was used to evoke APs by injecting 500 ms long depolarizing currents of increasing amplitude from –50 pA to 130 pA (Δ20 pA).

For sEPSC recordings, the internal solution consisted of 130 mM CsMeSO_4_, 10 mM CsCl, 4 mM NaCl, 10 mM HEPES, 1 mM MgCl_2_, 0.5 mM Na_3_GTP, 5 mM MgATP, 1 mM EGTA, 5 mM NMG, and 12 mM phosphocreatine. The cell voltage was held at −70 mV, and picrotoxin (100 μM) was added to the external ACSF.

sIPSCs were recorded using an internal solution consisting of 1 mM EGTA, 1 mM MgCl_2_, 100 mM CsCl, 5 mM MgATP, 10 mM HEPES, 0.5 mM Na_3_GTP, 30 mM NMG, and 12 phosphocreatine. The cell voltage was maintained at −70 mV, and recordings were performed in the presence of D-AP5 (50 μM) and DNQX (20 μM).

NMDAR-eEPSCs were recorded in the presence of picrotoxin (100 μM) and DNQX (20 μM) at a holding potential of 40 mV. The PPR was determined at –70 mV in the presence of picrotoxin (100 μM), and the interval between paired stimuli was set at 50 ms. The internal solution used was the same as that used for sEPSC recording.

### Golgi staining.

The mice were divided into the *Sh2d5^fl/fl^;EGFP* and *Sh2d5^fl/fl^;Cre* groups following transfection with different AAV-Cre vectors. The mice were sacrificed, and the brain tissue was removed. The brain tissues were washed with double-distilled water, and the neurons were stained with the Hito Golgi-Cox Best Staining Kit according to the manufacturer’s protocol. The sections were imaged with a microscope (Leica) at a magnification of 100× and analyzed with ImageJ software (NIH).

### Hippocampal neuronal culture and drug administration.

Primary neurons were obtained from the hippocampi of newborn *Sh2d5^fl/fl^* mice (P0–P1) and initially cultured in medium consisting of 10% FBS (Gibco), 1% penicillin-streptomycin (Gibco), and 89% DMEM/F12 (Gibco) for 4 hours. After this, the medium was replaced with complete medium consisting of 1% penicillin-streptomycin, 1% L-glutamate (Gibco), 2% B27 (Gibco), and 96% Neurobasal-A medium (Gibco). The cells were cultured for 3 days, followed by the addition of rAAV-*hSyn*-Cre-EGFP or rAAV-*hSyn*-EGFP.

After 7 additional days of culture to ensure that the cells reached the desired density and maturity, they were treated with CHX (MedChemExpress) (500 μM). Samples were collected at different time points (0, 2, 4, 6, 8, and 10 hours), and Western blotting was used to assess STAT1 degradation. Similarly, 7 days after AAV transfection, primary neurons were treated with KA, MG132 (a peptide-aldehyde proteasome inhibitor) (MedChemExpress), or CQ (MedChemExpress) at a concentration of 50 mM.

### Statistics.

Statistical analysis was performed using Graph Pad Prism 8 software. The experimental data are expressed as the medians and IQRs or means ± SEMs. The Supplemental Table 1 was expressed by mean ± SD. *P* < 0.05 was considered to indicate statistical significance. The Shapiro-Wilk test was used to test the normality of the data. If the variance of the data was significant, we used nonparametric tests for statistical analysis. 2-tailed Student’s *t* tests were used for comparisons between 2 independent groups, and 2-way ANOVA with Tukey test was used for comparisons among multiple groups.

### Study approval.

This study was conducted in accordance with the *Guide for the Care and Use of Laboratory Animals* (National Academies Press, 2011). All experiments were approved by the Animal Experimental Ethics Committee of Chongqing Medical University (ID: 2020-821).

The collection and use of all the human brain samples were approved by the Ethics Committee of the First Affiliated Hospital of Chongqing Medical University, and written informed consent was obtained from all patients in accordance with the Declaration of Helsinki.

### Data availability.

The IP-MS and LC-MS/MS data have been deposited in the ProteomeXchange Consortium (https://proteomecentral.proteomexchange.org) via the iProX partner repository with the dataset identifier PXD064659. All underlying raw data supporting the results described in the manuscript are provided in the Supporting Data Values file, with clearly labeled tabs corresponding to each figure panel.

## Author contributions

HG, HZ, CZ, and XT designed the study; HG, HZ, and CZ performed the experiments; YS, LJ, and MY participated in data and sample collection; YZ, NZ, RZ, and RY analyzed the data; HG, YY, and XT wrote the manuscript; and XT, and YY supervised the project. All the authors read and approved the final paper.

## Supplementary Material

Supplemental data

Unedited blot and gel images

Supporting data values

## Version 1 08/22/2025

Electronic publication
