# Supplementary material for: Deletion of SH2D5 alleviates epileptic seizures and NMDAR expression via autophagic degradation of STAT1
Source: JCI Insight. 2025 Aug 22;10(16):e191347. doi: 10.1172/jci.insight.191347 (PMC12406720; doi:10.1172/jci.insight.191347)
Supplement: Supplemental data [file jciinsight-10-191347-s181.pdf]

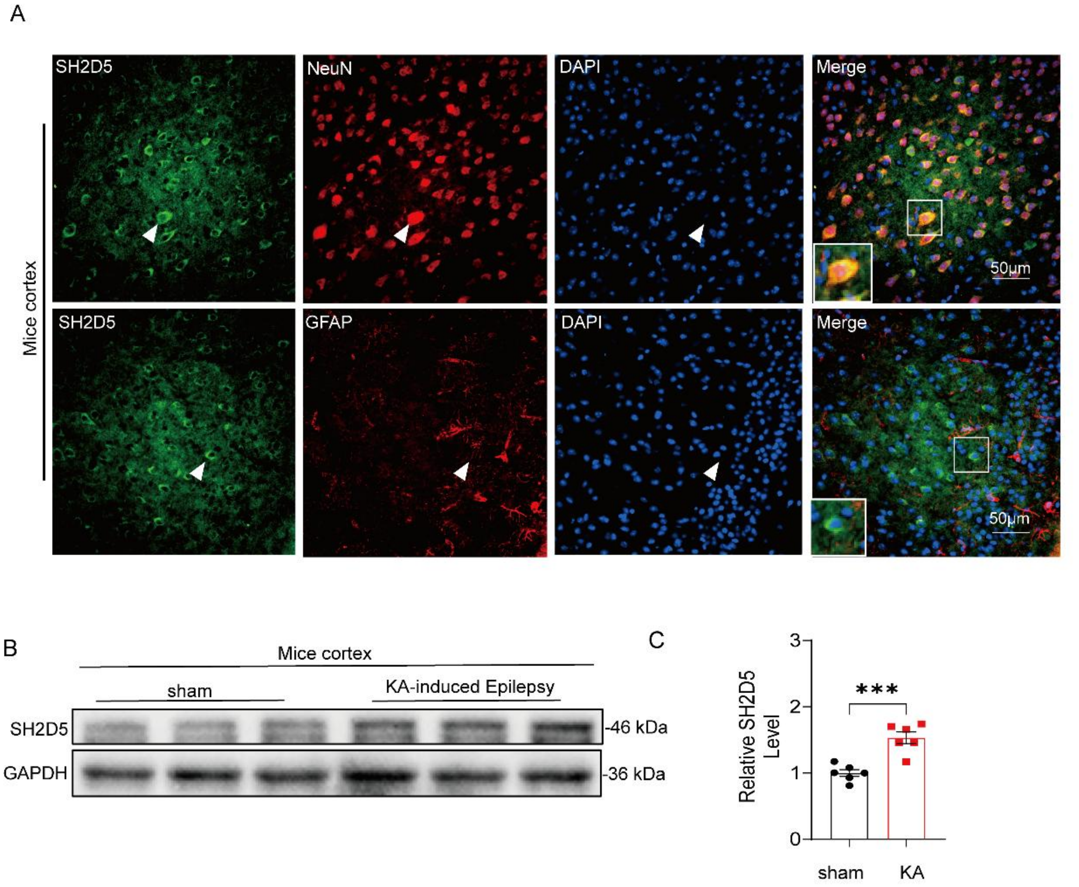

Supplementary Figure 1. SH2D5 expression is upregulated in the cortices of KA-induced epilepsy model mice

(A) Immunofluorescence staining of SH2D5 (green) in the mouse cortex. Costaining for NeuN (red, upper panel) or GFAP (red, lower panel) and DAPI (blue, nuclei) revealed SH2D5 expression in neurons. The merged images highlight colocalization (yellow), with magnified insets showing specific instances of colocalization. Scale bar = 50 μm. (B) Western blot analysis of SH2D5 protein levels in the cortices of sham mice and KA-induced epilepsy model mice. (C) Quantification of SH2D5 protein levels normalized to those of GAPDH levels in the sham and KA groups. The data are presented as the mean ± SEM. Unpaired t test. ***$P < 0.001$

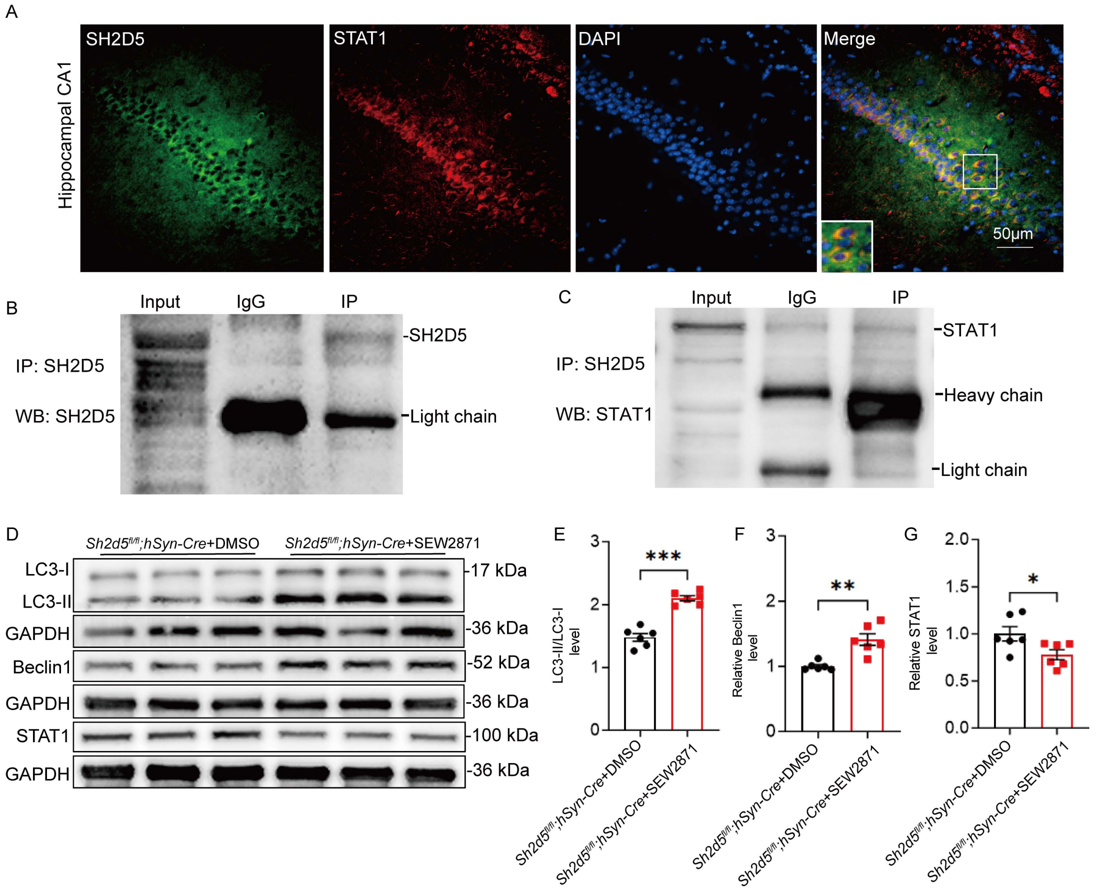

Supplementary Figure 2. Interaction between SH2D5 and STAT1 and its impact on the Rac1 pathway

(A) Immunofluorescence staining of the mouse hippocampal CA1 region showing the localization of SH2D5 (green) and STAT1 (red); DAPI (blue) was used to label nuclei. Yellow signals in the merged images (Merge) indicate the colocalization of SH2D5 and STAT1, and the boxed areas are magnified. Scale bar = 50 μm. (B-C) In the left panel, immunoprecipitation with an anti-SH2D5 antibody followed by immunoblotting for SH2D5 was used to detect SH2D5; in the right panel, immunoprecipitation with an anti-SH2D5 antibody followed by immunoblotting for STAT1 was used to detect STAT1. Representative Western blot images (D) and quantification (E-G) of LC-II /LC-I, Beclin1, and STAT1 levels after SEW2871 administration. The data are presented as the means ± SEMs. Unpaired t test. **P* < 0.05, ***P* < 0.01, and ****P* < 0.001; n.s., no significant difference.

Supplementary Table 1 Clinical characteristics of TLE patients and TBI patients.

group	age（year）	Gender (M/F)	Course(years)	AEDs before surgery	Resection tissue	Neuropathological diagnosis
TBI1	21	F	0	None	TN, L	N
TBI2	38	F	0	None	TN, R	N
TBI3	28	M	0	None	TN, R	N
TBI4	31	F	0	None	TN, R	N
TBI5	26	M	0	None	TN, R	N
TBI6	25	M	0	None	TN, L	N
TLE1	19	F	10	VPA, CBZ, CZP, PB	TN, L	NL, G
TLE2	37	M	18	VPA, TPM, CBZ	TN, R	NL, G
TLE3	22	M	13	VPA, CBZ, PB, TPM	TN, L	NL, G
TLE4	22	F	9	VPA, CBZ, TPM	TN, R	NL, G
TLE5	37	M	14	VPA, CBZ, PHT, TPM	TN, R	NL, G
TLE6	21	F	11	VPA, OXC, LTG	TN, L	NL, G

AEDs, antiepileptic drugs; CBZ, carbamazepine; CZP, clonazepam; F, female; G, gliosis; L, left; LTG, lamotrigine; M, male; N, relative normal; NL, neuron loss; OXC, oxcarbazepine; PB, phenobarbital; PHT, phenytoin; R, right; TBI, traumatic brain injury; TLE, temporal lobe epilepsy; TN, temporal neocortex; TPM, topiramate; VPA, valproate.

Sample	TBI group (n = 6)	TLE group (n = 6)	P values
Age (years)	26± 5	27 ± 9	0.6685
Female / Male ratio	3/3	3/3	1

P values were calculated using two-tailed Student's t-test (age) or a Pearson Chi-square test (Female / Male ratio). $P < 0.05$ was considered statistically significant, and the experimental data were expressed by mean ± standard deviation.

Supplementary Table 2 Information on PCR primers for relevant genes

RNAprimer of qPCR

Gene name	Forward primer	Reverse primer
Mouse Gapdh	5'-CAGTGGCAAAGTGGAGATTGTTG-3'	5'-TCGCTCCTGGAAGATGGTGAT-3'
Mouse Stat1	5'-CACCTATGAGCCCGACCCTATTA-3'	5'-TTTCACCAACAGTCTCAGCTTGA-3'
Mouse Glua1	5'-GTCAGCCGTTTCAGTCCTTATGA-3'	5'-GTTGGCTGTGTATGAGGAGATGA-3'
Mouse Glua2	5'-CGTGTAATCCTTGACTGCGAAAG-3'	5'-TCAGCAGGTCTCCATCAGTAAAT-3'
Mouse Glun1	5'-AGTTTGACCCAGGAACCAAGAAT-3'	5'-GACTCGTTCTTGCCGTTGATTAG-3'
Mouse Glun2a	5'-GCTACACACTCTGCACCAATTTA-3'	5'-TTTCCCATTCCCGGTCCTTATTC-3'
Mouse Glun2b	5'-ATGCATCCGAAGCTGGTGATAAT-3'	5'-CACATTCGAGGCCACACATAGTA-3'

Verification of SH2D5 transgenic mice

primer 5'-CAGCTGCAGGCCTCTTACAT-3' 5'-GCTGCAGACACGAAAGTCAG-3'
