# Supplementary material for: Deletion of SH2D5 alleviates epileptic seizures and NMDAR expression via autophagic degradation of STAT1
Source: JCI Insight. 2025 Aug 22;10(16):e191347. doi: 10.1172/jci.insight.191347 (PMC12406720; doi:10.1172/jci.insight.191347)

Figure 1C.

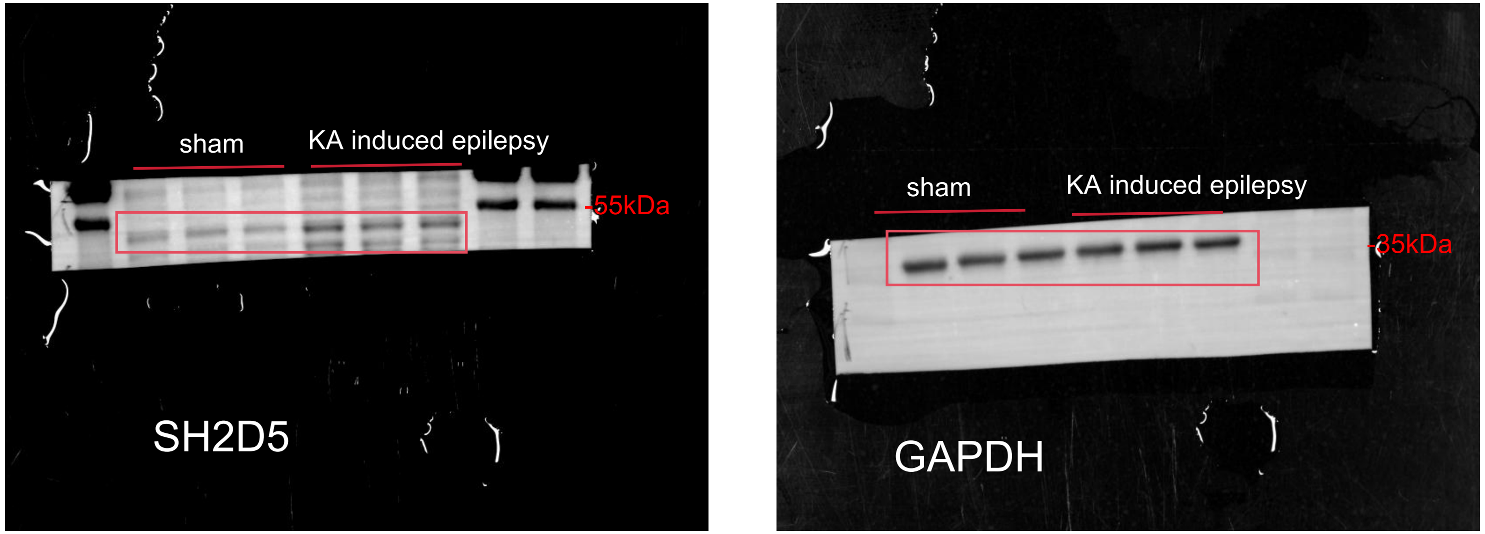

Figure 1D.

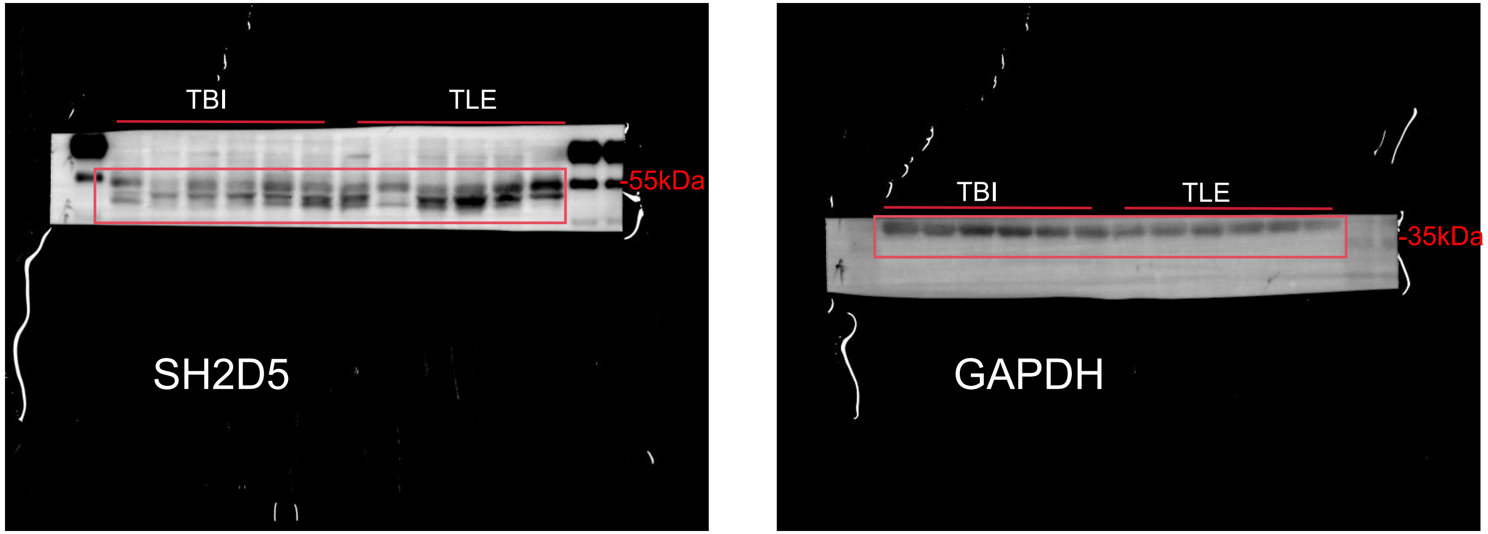

Figure 2D.

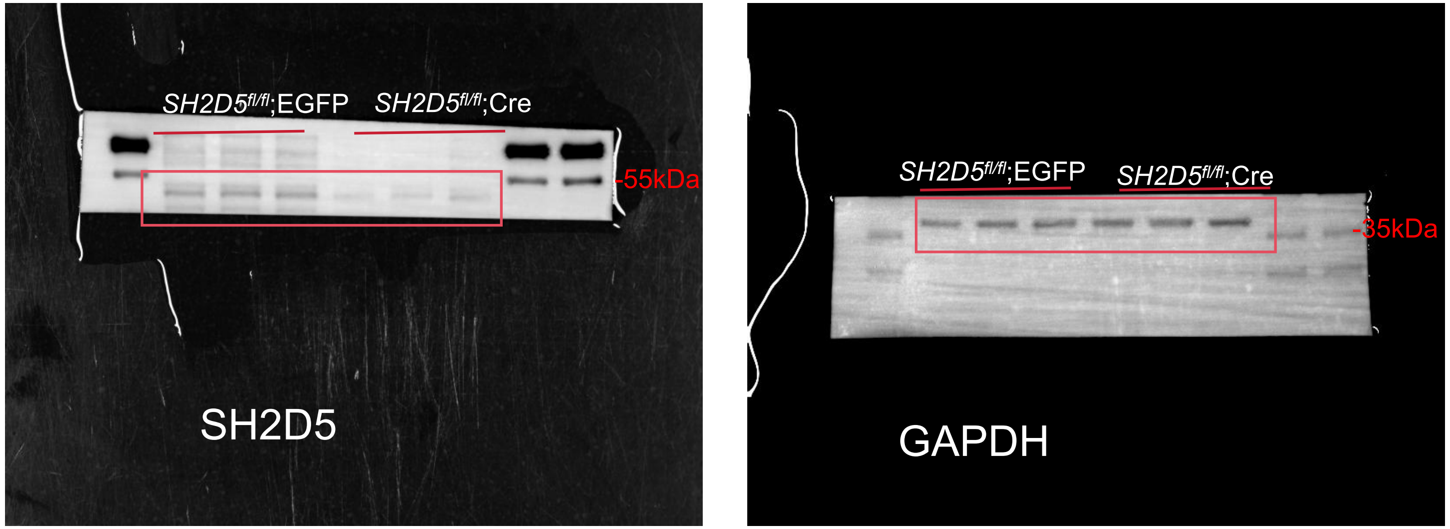

Figure 5C.

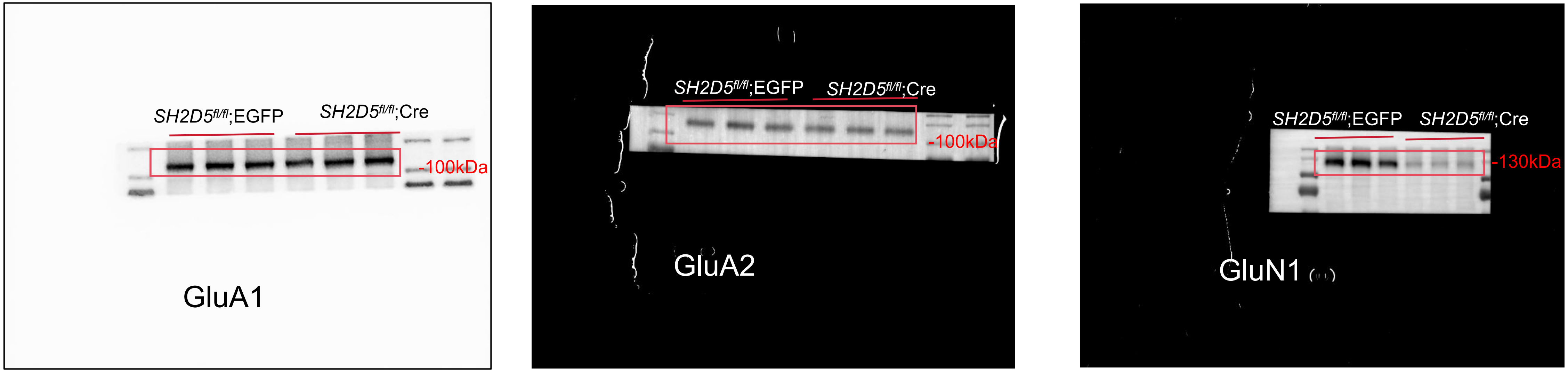

Figure 5C.

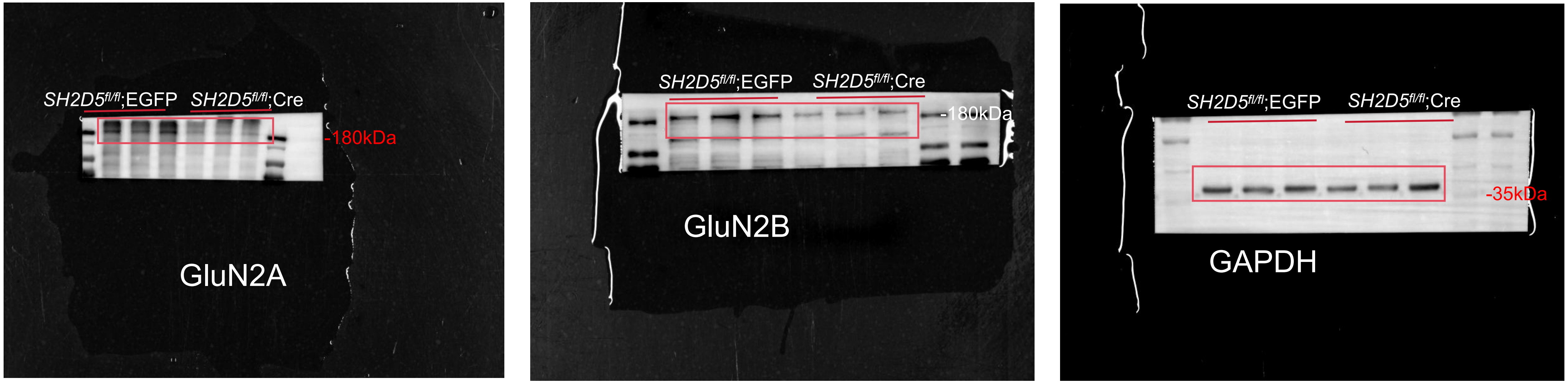

Figure 5E.

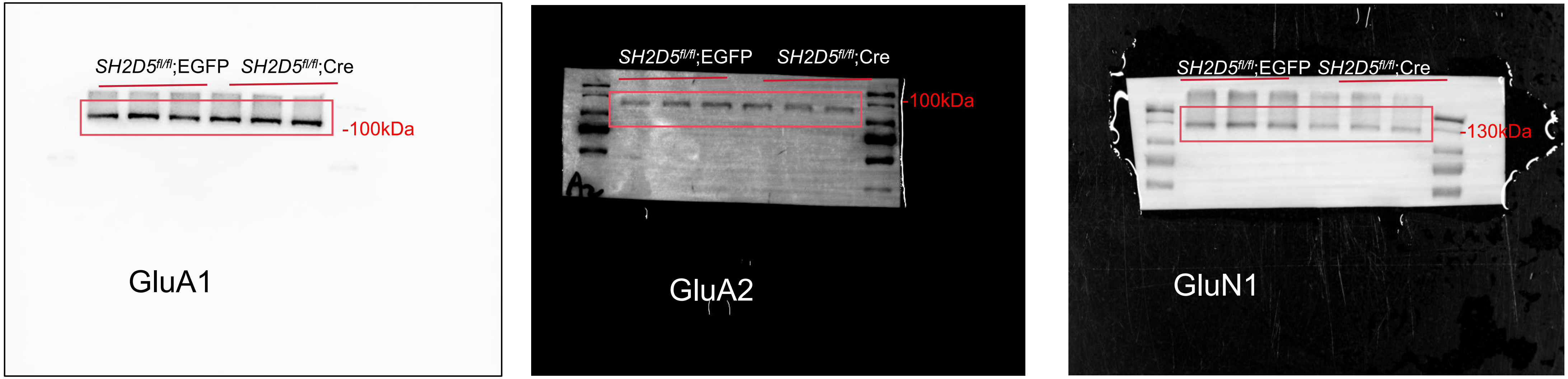

Figure 5E.

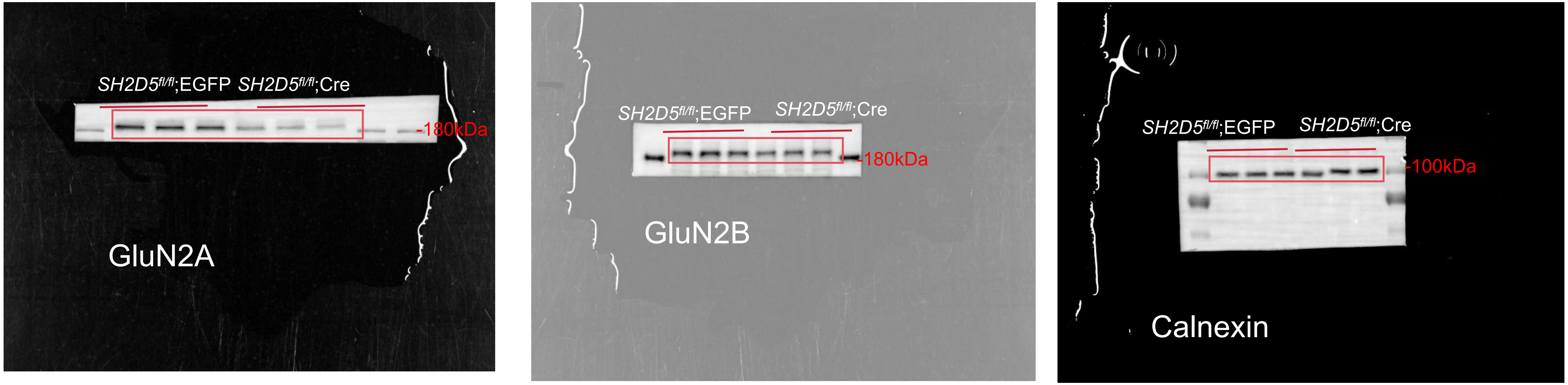

Figure 6D.

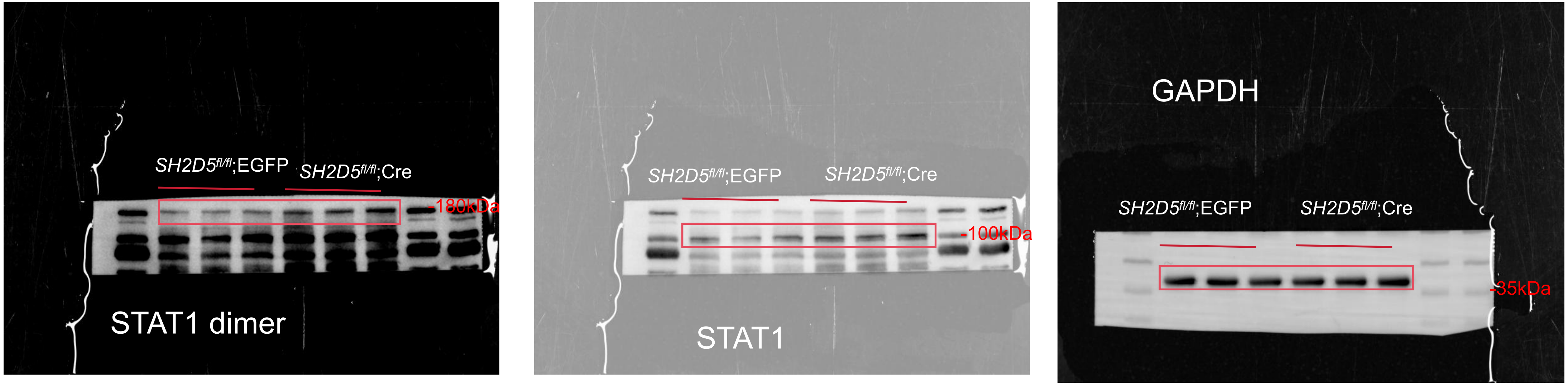

Figure 6F.

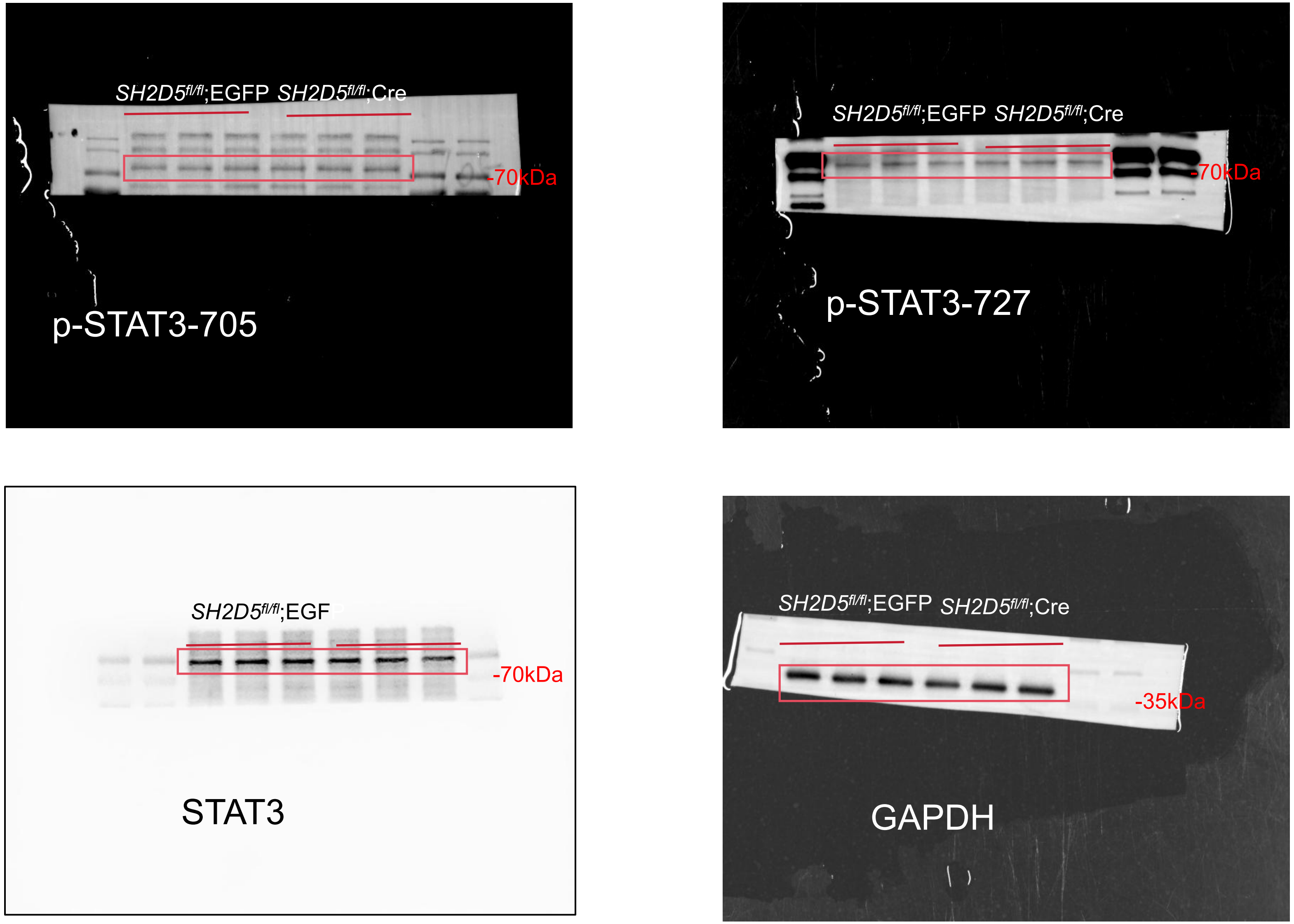

SH2D5$^{fl/fl}$;EGFP SH2D5$^{fl/fl}$;Cre
-70kDa
p-STAT3-705
SH2D5$^{fl/fl}$;EGFP SH2D5$^{fl/fl}$;Cre
-70kDa
p-STAT3-727
SH2D5$^{fl/fl}$;EGF
-70kDa
STAT3
SH2D5$^{fl/fl}$;EGFP SH2D5$^{fl/fl}$;Cre
-35kDa
GAPDH

Figure 6H.

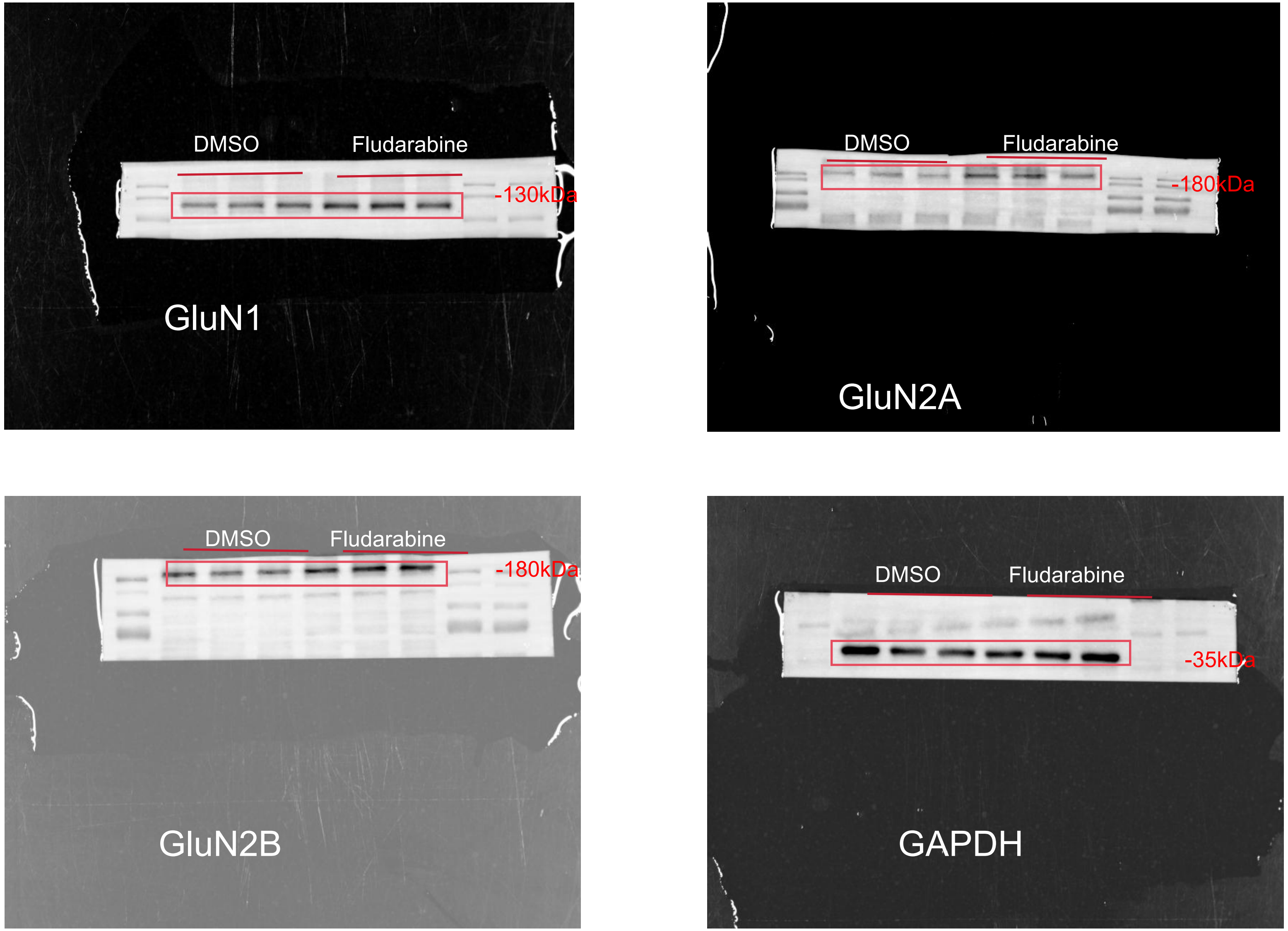
DMSO
Fludarabine
-130kDa
GluN1
DMSO
Fludarabine
-180kDa
GluN2A
DMSO
Fludarabine
-180kDa
GluN2B
DMSO
Fludarabine
-35kDa
GAPDH

Figure 7B.

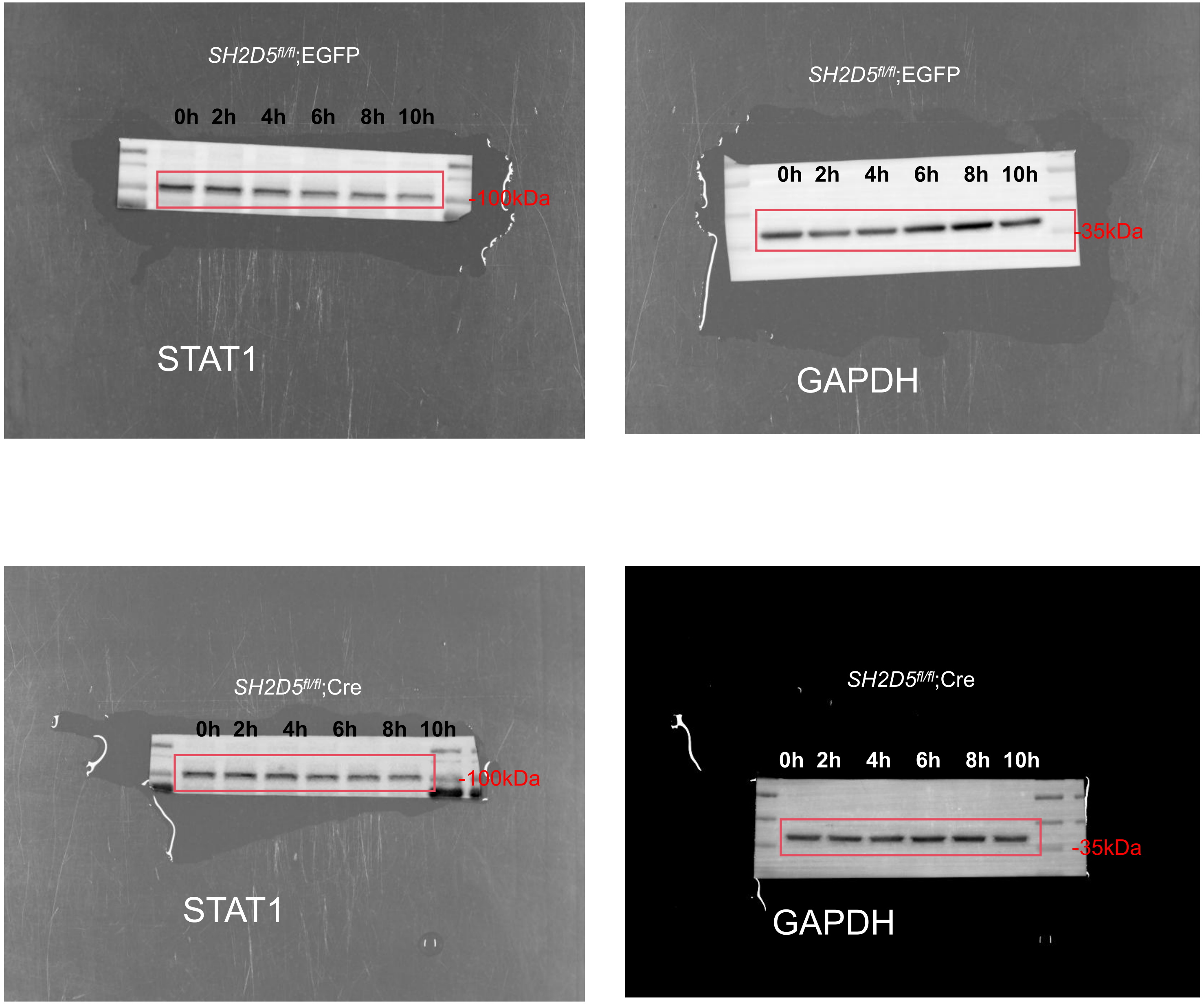

SH2D5fl/fl;EGFP
0h 2h 4h 6h 8h 10h
-100kDa
STAT1
SH2D5fl/fl;EGFP
0h 2h 4h 6h 8h 10h
-35kDa
GAPDH
SH2D5fl/fl;Cre
0h 2h 4h 6h 8h 10h
-100kDa
STAT1
SH2D5fl/fl;Cre
0h 2h 4h 6h 8h 10h
-35kDa
GAPDH

Figure 7D.

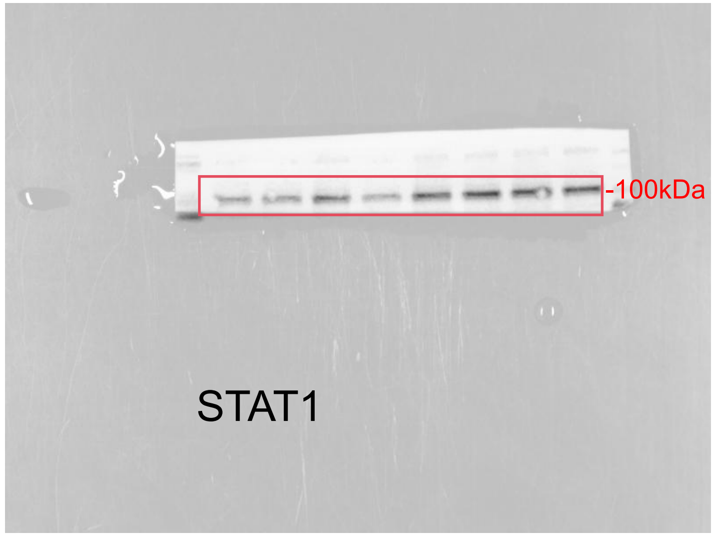

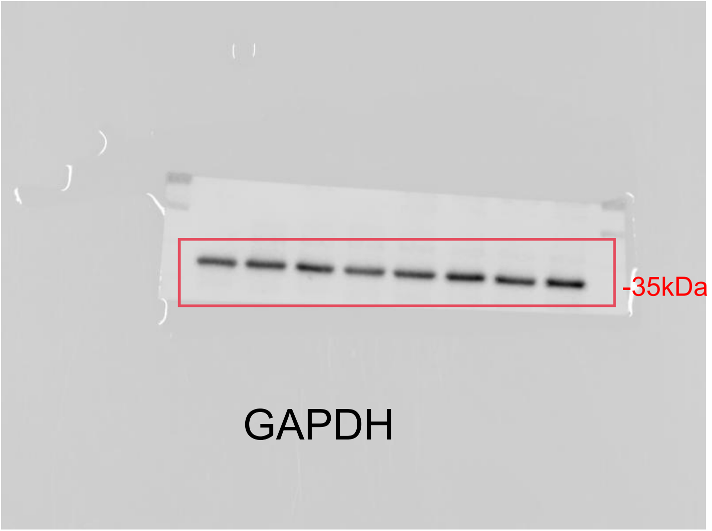

Figure 7E.

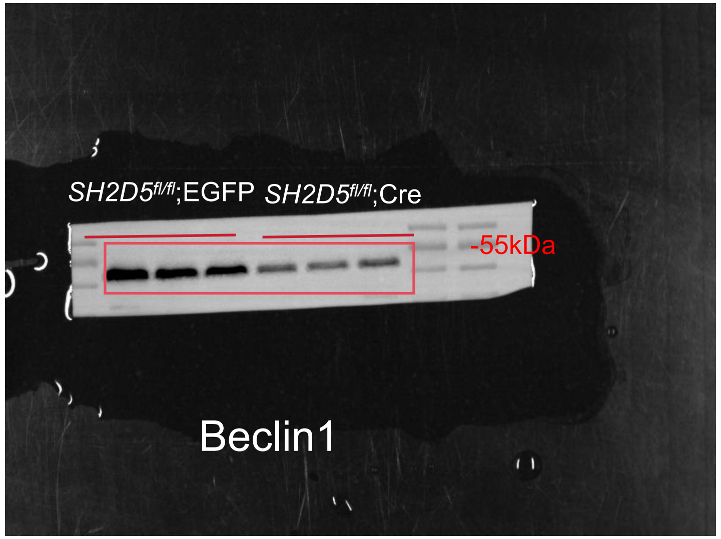

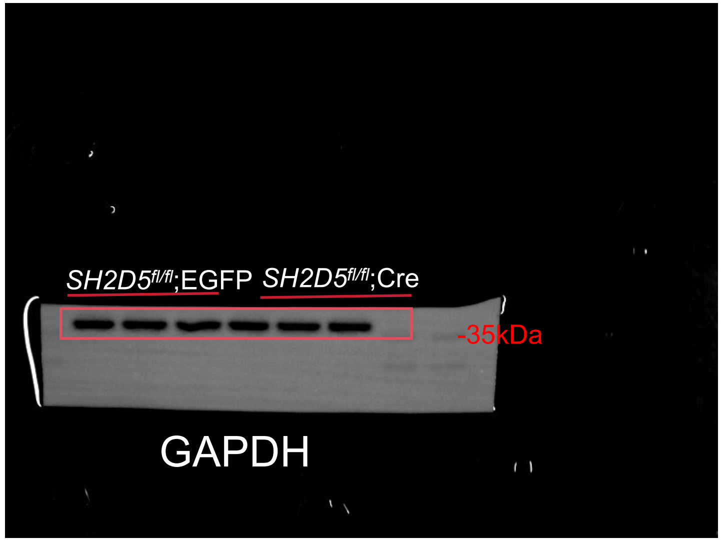

Figure 7H.

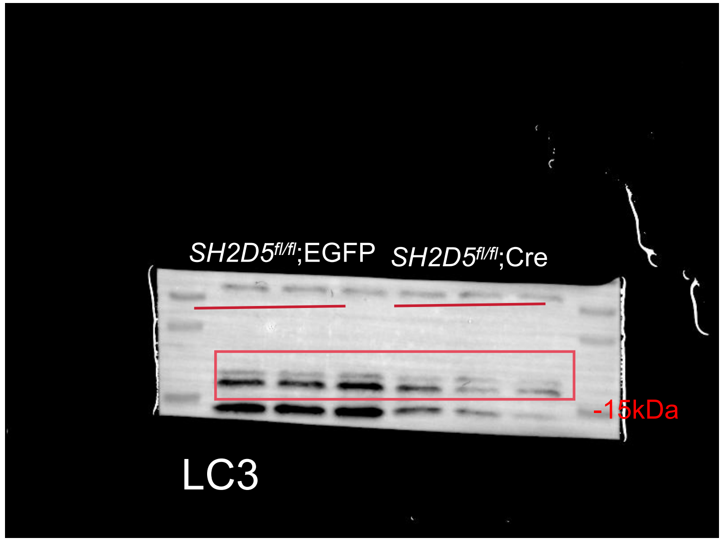

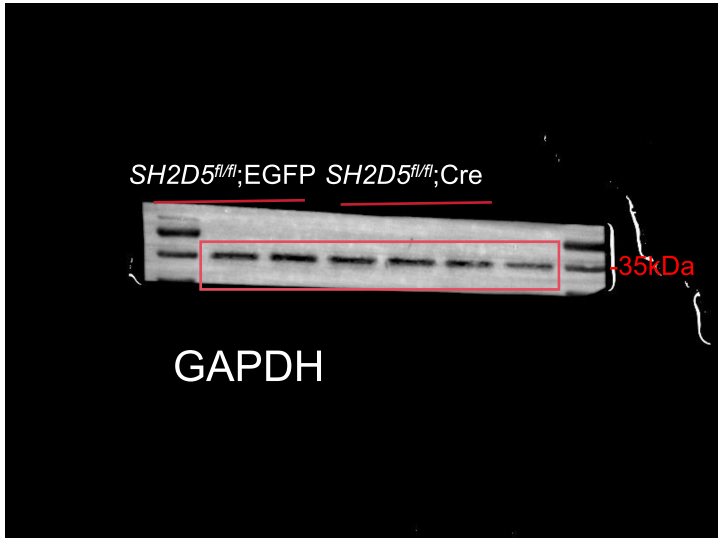

Figure 7J.

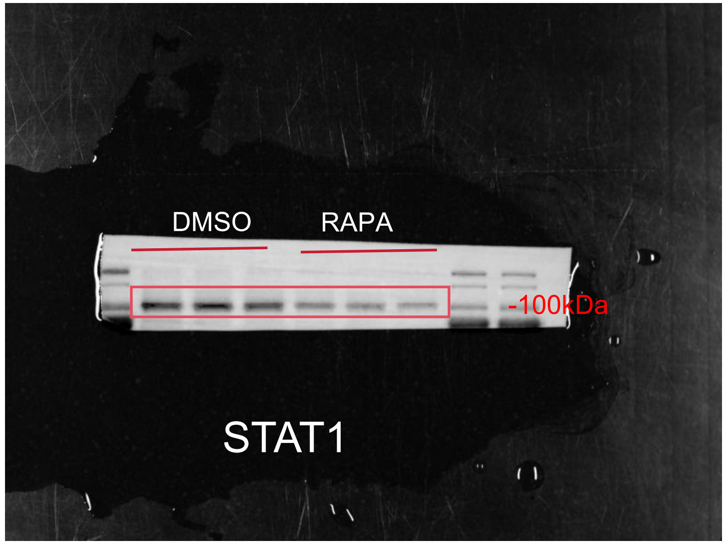

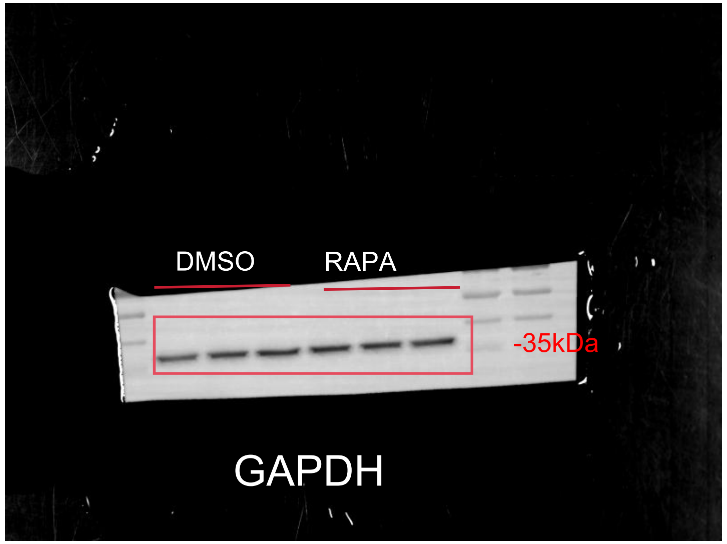

Figure 7L.

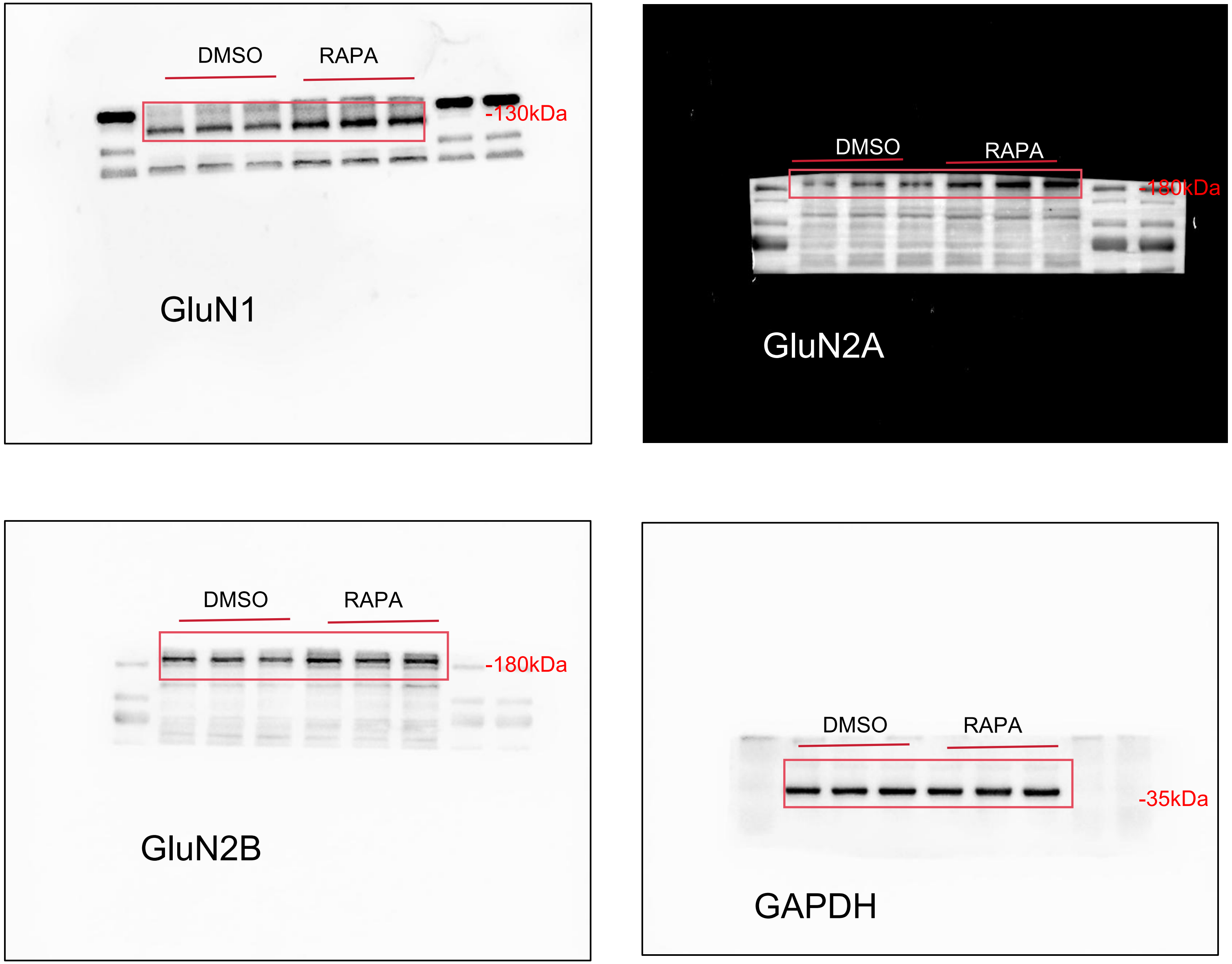
DMSO
RAPA
-130kDa
GluN1
DMSO
RAPA
-180kDa
GluN2A
DMSO
RAPA
-180kDa
GluN2B
DMSO
RAPA
-35kDa
GAPDH

Supplementary Figure 1B.

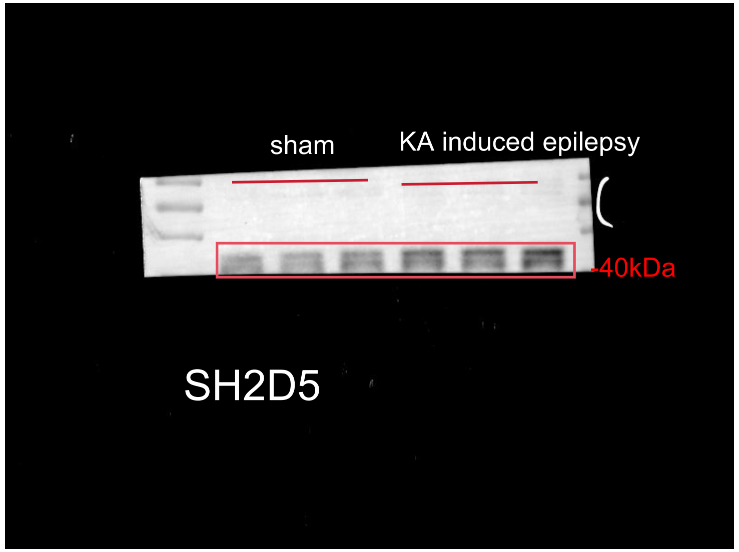

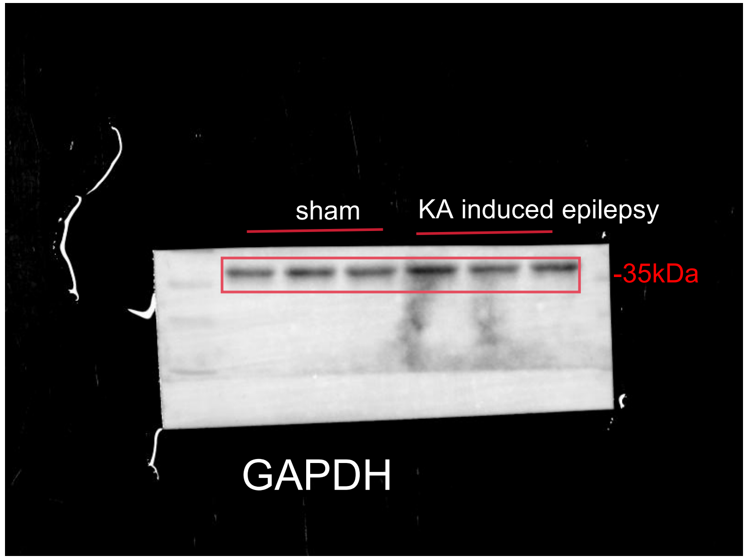

Supplementary Figure 2B.

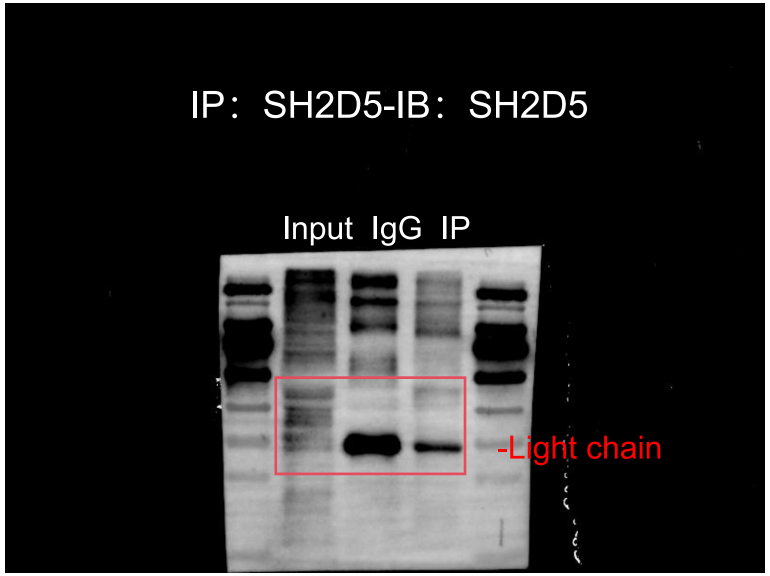

Supplementary Figure 2C.

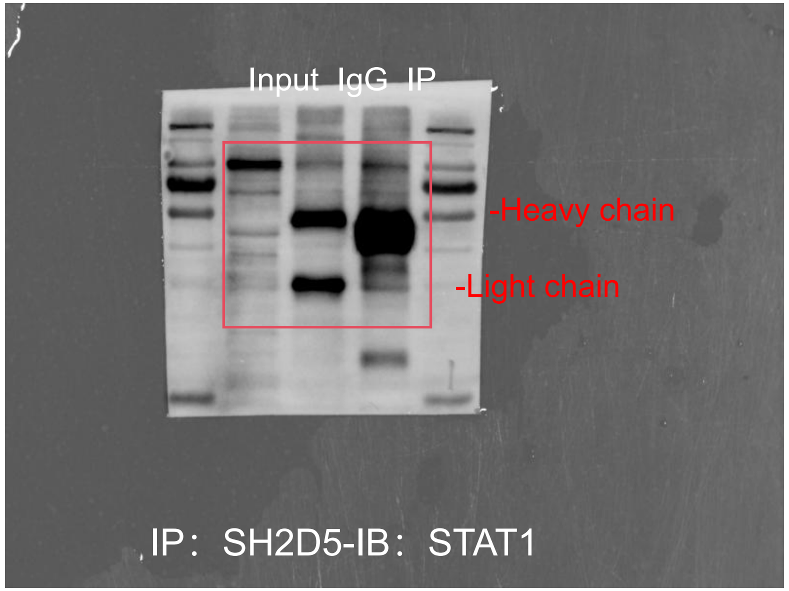

Supplementary Figure 2D.

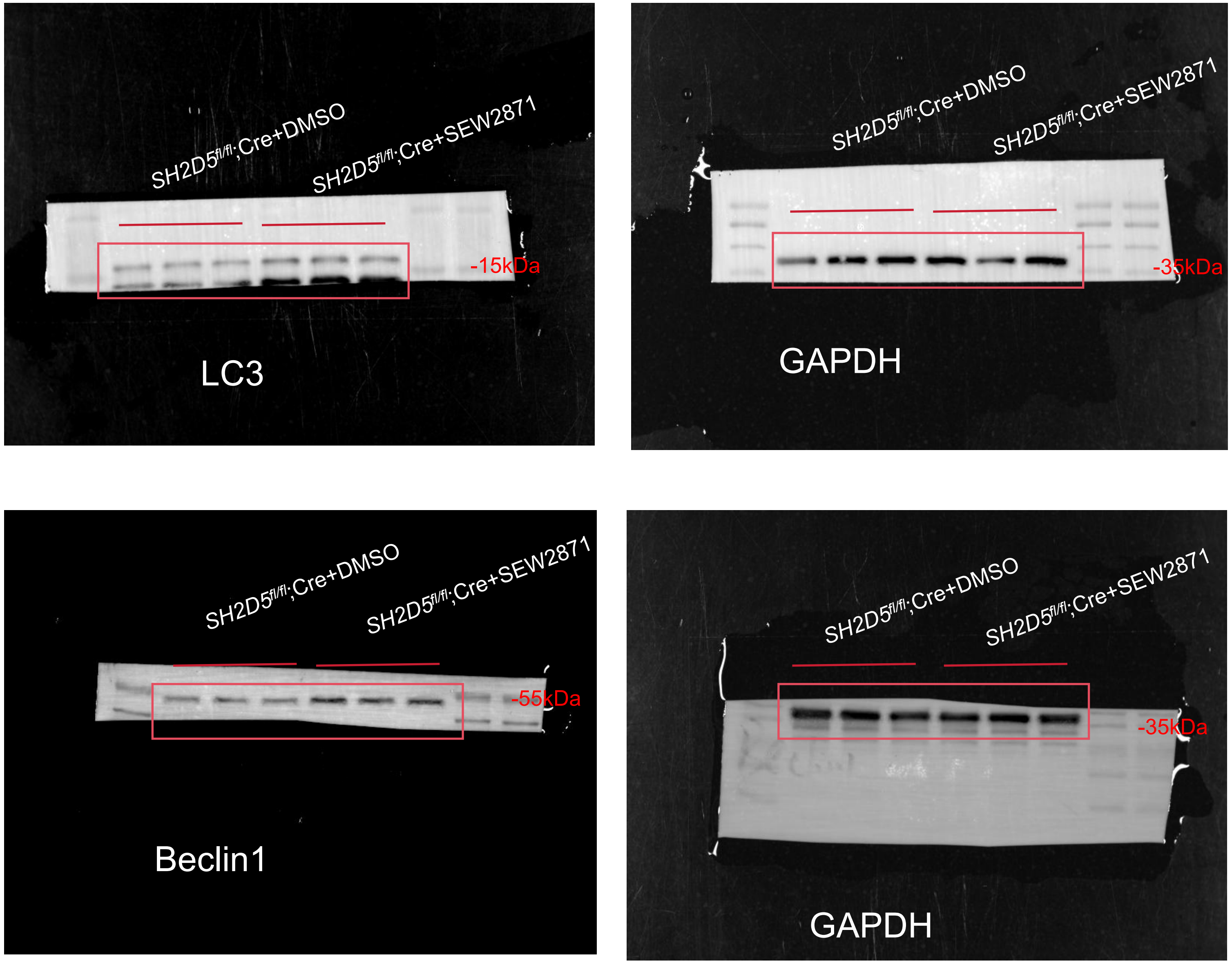

Supplementary Figure 2D.

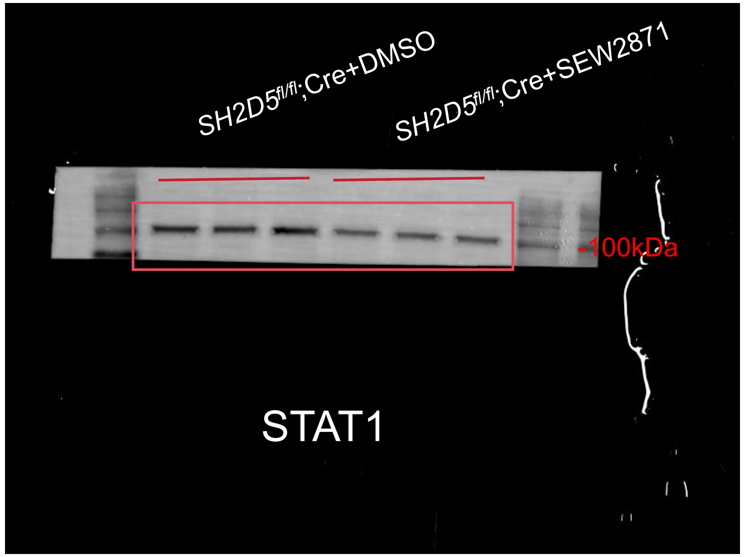

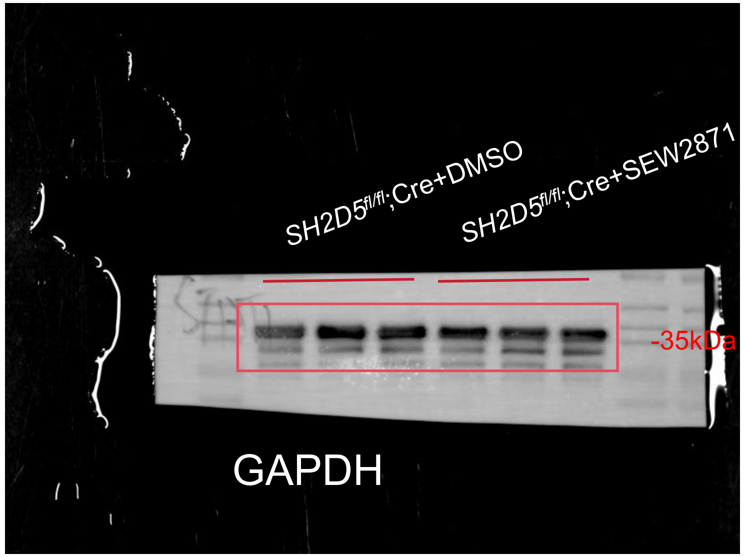

Supplement: Unedited blot and gel images [file jciinsight-10-191347-s182.pdf]
